# A Single Amino Acid Deletion (ΔF1502) in the S6 Segment of Ca_V_2.1 Domain III Associated with Congenital Ataxia Increases Channel Activity and Promotes Ca^2+^ Influx

**DOI:** 10.1371/journal.pone.0146035

**Published:** 2015-12-30

**Authors:** Maria Isabel Bahamonde, Selma Angèlica Serra, Oliver Drechsel, Rubayte Rahman, Anna Marcé-Grau, Marta Prieto, Stephan Ossowski, Alfons Macaya, José M. Fernández-Fernández

**Affiliations:** 1 Laboratori de Fisiologia Molecular i Canalopaties, Departament de Ciències Experimentals i de la Salut, Universitat Pompeu Fabra, Barcelona, Spain; 2 Genomic and Epigenomic Variation in Disease Group, Centre for Genomic Regulation (CRG), The Barcelona Institute of Science and Technology, Barcelona, Spain; 3 Universitat Pompeu Fabra, Barcelona, Spain; 4 Pediatric Neurology Research Group, Vall d’Hebron Research Institute, Universitat Autònoma de Barcelona, Barcelona, Spain; Dalhousie University, CANADA

## Abstract

Mutations in the *CACNA1A* gene, encoding the pore-forming Ca_V_2.1 (P/Q-type) channel α_1A_ subunit, result in heterogeneous human neurological disorders, including familial and sporadic hemiplegic migraine along with episodic and progressive forms of ataxia. Hemiplegic Migraine (HM) mutations induce gain-of-channel function, mainly by shifting channel activation to lower voltages, whereas ataxia mutations mostly produce loss-of-channel function. However, some HM-linked gain-of-function mutations are also associated to congenital ataxia and/or cerebellar atrophy, including the deletion of a highly conserved phenylalanine located at the S6 pore region of α_1A_ domain III (ΔF1502). Functional studies of ΔF1502 Ca_V_2.1 channels, expressed in *Xenopus* oocytes, using the non-physiological Ba^2+^ as the charge carrier have only revealed discrete alterations in channel function of unclear pathophysiological relevance. Here, we report a second case of congenital ataxia linked to the ΔF1502 α_1A_ mutation, detected by whole-exome sequencing, and analyze its functional consequences on Ca_V_2.1 human channels heterologously expressed in mammalian tsA-201 HEK cells, using the physiological permeant ion Ca^2+^. ΔF1502 strongly decreases the voltage threshold for channel activation (by ~ 21 mV), allowing significantly higher Ca^2+^ current densities in a range of depolarized voltages with physiological relevance in neurons, even though maximal Ca^2+^ current density through ΔF1502 Ca_V_2.1 channels is 60% lower than through wild-type channels. ΔF1502 accelerates activation kinetics and slows deactivation kinetics of Ca_V_2.1 within a wide range of voltage depolarization. ΔF1502 also slowed Ca_V_2.1 inactivation kinetic and shifted the inactivation curve to hyperpolarized potentials (by ~ 28 mV). ΔF1502 effects on Ca_V_2.1 activation and deactivation properties seem to be of high physiological relevance. Thus, ΔF1502 strongly promotes Ca^2+^ influx in response to either single or trains of action potential-like waveforms of different durations. Our observations support a causative role of gain-of-function Ca_V_2.1 mutations in congenital ataxia, a neurodevelopmental disorder at the severe-most end of *CACNA1A*-associated phenotypic spectrum.

## Introduction

The Ca_V_2.1 (P/Q-type) Ca^2+^ channel, encoded by the *CACNA1A* gene, is a high-voltage-activated channel composed of a main transmembrane pore-forming subunit (α_1A_), associated to a disulfide-linked α_2_δ subunit dimer, and one or more intracellular β subunits (Ca_V_β_1–4_). The α_1A_ subunit consists of four repeated domains (I–IV) each containing six transmembrane regions (S1–S6) with a voltage sensor (S1–S4) and a pore region (S5, P-loop and S6). The N- and C-terminal regions as well as the large intracellular loops between α_1A_ domains serve as signalling platforms for channel gating regulation processes, which includes the functional interaction with presynaptic proteins such as SNARE proteins of the vesicle docking/fusion machinery [1,2]. Indeed, Ca_V_2.1 channels are localized at presynaptic terminals [3] tightly coupled to neurotransmitter release [1]. Nevertheless, it also presents somatodendritic localization in neurons, thus contributing to the generation of postsynaptic responses, such as neuronal excitability [4–6], gene expression [7], Ca^2+^ signaling microdomains [8], and neuronal survival [9].

Ca_V_2.1 channels are expressed in all brain areas involved in the pathogenesis of migraine, including the cerebral cortex and nociceptive pathways, and their expression is particularly high in the cerebellum (reviewed in [10,11]). Accordingly, mutations in the *CACNA1A* gene are linked to familial and sporadic hemiplegic migraine (FHM/SHM), and both episodic and progressive forms of ataxia (reviewed in [11]).

FHM and SHM are rare subtypes of migraine with aura that include hemiparesis during the aura phase [10–12]. Hemiplegic Migraine (HM) mutations in the α_1A_ channel subunit locate around the line pore (S5-P-S6 segments), in the loops connecting S3-S4 or S4-S5 segments and in the S1 and S4 segments of the voltage sensors [11,13,14]. The functional analysis of all HM *CACNA1A* mutations evaluated so far, both in heterologous expression systems and excitatory neurons from FHM knock-in (KI) mice, revealed an overall gain of Ca_V_2.1 channel function, mainly due to a reduction in the voltage threshold of channel activation [11,15–18]. Further studies in FHM KI mice indicate that such gain-of-channel-function specifically enhances excitatory transmission at cortical synapses to favor initiation and propagation of cortical spreading depression (CSD), an abnormal increase of cortical activity -followed by a long-lasting neuronal suppression wave- that propagates across the cortex and triggers the aura and migraine itself [10–12,16–20].

Episodic ataxia type 2 (EA2), is a rare neurological disorder, typically with childhood onset (although late-onset EA2 has been also reported [21]), characterized by paroxysmal attacks (lasting hours to days) of cerebellar dysfunction which are commonly triggered by emotional and physical stress. Some patients may exhibit mild interictal cerebellar ataxia or isolated nystagmus or, more rarely, severe progressive ataxia [11]. EA2 is caused by *CACNA1A* mutations that mostly result in truncation of the encoded Ca_V_2.1 α_1A_ channel subunit, while missense mutations have also been reported [22]. Although most of the missense mutations are located in the pore regions of the channel, there are at least five mutations of highly conserved residues found in the voltage sensor domains [11,23–25]. Usually, these mutations have negative effects on both channel activity and trafficking to the plasma membrane when studied in heterologous expression systems [11,23–26]. At present it is unknown whether aberrant trafficking of Ca_V_2.1 EA2 mutants also occurs in patients’ neurons. It has been reported that the spontaneous ataxic mouse model rolling Nagoya (carrying Ca_V_2.1 R1262G mutation at S4- DIII) has reduced voltage sensitivity and activity of the Ca_V_2.1 channel but shows unaltered expression in mouse cerebellar Purkinje cells in contrast to heterologously expressed R1262G channels showing strongly reduced expression and/or targeting to the plasma membrane [27]. Therefore, other channel function modifications beyond the decreased intracellular trafficking may lie beneath the pathophysiological role of Ca_V_2.1 in EA2 patients. In this sense, it has been reported that several Ca_V_2.1 EA2 mutations (affecting either pore or voltage sensor regions) produce loss-of-channel-function by shifting channel activation to more depolarized voltages [25,26,28–31], thus reducing Ca_V_2.1 open probability [31]. Besides, A454T mutation (located at the first intracellular loop connecting domains I and II of α_1A_) has been linked to a heterogeneous ataxic disorder with clinical features midway between EA2 and spinocerebellar ataxia type 6 (SCA6, other allelic disorder associated with mutations in the *CACNA1A* gene) [32]. Mutation A454T also produces loss-of-channel function by altering the functional interaction between α_1A_ and SNARE proteins thus leading to a decreased channel coupling to exocytosis [2]. Regardless of the mechanism employed by Ca_V_2.1 EA2 mutations, studies derived from an EA2 KI mouse model (carrying the loss-of-function pore mutation F1406C) suggest that motor deficit apparently results from subtle dysfunction of multiple cerebellar cell types [33].

SCA6 is a late-onset slowly progressive ataxic syndrome with underlying cerebellar atrophy. It is caused by small expansions of a polyglutamine (polyQ) sequence, encoded by CAG trinucleotide repeats, at the C-terminal end of some Ca_V_2.1 splice variants [34]. Studies in SCA6 KI mice show that the polyQ expansions do not alter Ca_V_2.1 biophysical properties and suggest that the markedly selective degeneration of cerebellar Purkinje cells (and the subsequent motor impairment) is due to the toxic accumulation of the expanded polyQ channel [11,35].

Several HM-linked *CACNA1A* gain-of-function mutations also produce permanent cerebellar symptoms ranging from slowly progressive cerebellar ataxia and/or nystagmus (with cerebellar atrophy in some cases) [14,36,37] or permanent ataxia [38,39] to early-onset cerebellar signs consistent with congenital ataxia [40–44]. In an attempt to understand how a *CACNA1A* HM mutation can also lead to such severe clinical phenotype, the deletion of a highly conserved phenylalanine located at the S6 pore region of α_1A_ domain III (ΔF1502), found in a case of congenital ataxia and hemiplegic migraine, has recently been functionally characterized [44]. The study, performed in *Xenopus* oocytes as heterologous expression system, only revealed discrete alterations in Ca_V_2.1 channel function of unclear pathophysiological relevance, since channel activity was measured with Ba^2+^ as the charge carrier and not with the physiological permeant ion Ca^2+^. The most relevant detected effect was an ~ 11 mV hyperpolarizing shift of the voltage-dependent channel activation, similar to the one reported for all HM mutations analyzed so far (including those that do not lead to cerebellar symptoms) [13,14] (for a review see [11]). Such gain-of-function effect fits well with a pathophysiological role of mutation ΔF1502 in HM but it seems not enough to explain its involvement in congenital ataxia *per se* [44]. Here, we analyze the functional consequences of the ΔF1502 α_1A_ mutation (found in a second case of congenital ataxia) on Ca_V_2.1 human channels heterologously expressed in mammalian tsA-201 HEK cells, using the physiological ion Ca^2+^ as the charge carrier. Our results reveal that ΔF1502 is a strong gain-of-function mutation, as it powerfully decreases the voltage threshold for channel activation (by ~ 21 mV). This effect, in combination with ΔF1502-induced changes in the kinetics of activation, deactivation and inactivation of Ca_V_2.1 channels, allow significantly higher Ca^2+^ influx in response to stimuli of physiological traits, and support the causative role of ΔF1502 (and, by extension, of other gain-of-function Ca_V_2.1 mutations) in congenital ataxia and/or cerebellar atrophy.

## Materials and Methods

### Brain Magnetic Resonance Imaging (MRI) Protocol

MR data were acquired using a 1.5 T scanner (MAGNETOM Symphony or MAGNETOM Vision, Siemens, Erlangen, Germany) equipped with a circular polarized receiver head array coil. Sagittal, transverse and coronal conventional spin-echo T1-weighted sequences were obtained (repetition time [TR] /echo time [TE]/acquisitions 450–600 ms/12-20 ms/2). In addition, transverse T2-weighted fast spin-echo (TR 4300 ms/TE 96 ms/acquisitions 1–2) and fast-FLAIR (TR 8500 ms/ TE 104 ms/ inversion time 2500 ms/acquisitions1) were also performed. All sequences were obtained with 4-5-mm section thickness and .1-.3 interslice gap, 144–256 × 256–384 matrix, and 196 × 230 mm field of view.

### Genetic Analyses

#### Sample preparation and sequencing

DNA from the trio (patient and parents) was extracted from blood following a standard salting-out protocol. An ultra sound device (Covaris, Woburn, MA, USA) was used for shearing of 1μg of genomic DNA aiming for a fragment size of 300-400bp. Library generation was performed using the TruSeq DNA kit (Illumina, San Diego, CA, USA) following supplier’s instructions. Pools of six samples were subjected to whole exome capture applying Nimblegene SeqCap EZ Human Exome version 3 (Roche NimbleGen, Madison, WI, USA) followed by sequencing on a HiSeq 2500 using the 2x100bp protocol. We obtained a coverage of at least 30-fold in > 80% of the probes.

#### Alignment and variant detection

Sequencing data preparation, read alignment and variant prediction was performed according to the ‘GATK best practice’ recommendations for a small number of samples [45]. In brief, sequencing reads were aligned to the hg19 reference genome using BWA-MEM (version 0.7.7) [46], followed by GATK indel realignment and base quality recalibration. Variants were predicted using GATK UnifiedGenotyper (version 3.1.1) (SNVs) and ClinDel (Indels) [47] in regions covered by SeqCap probes extended by 150 nt.

#### Variant annotation and prioritization

Identified genetic variants were subjected to annotation using ANNOVAR [48]. In addition information on population allele frequencies (1000 genomes, Exome Variant Server, dbSNP), damage estimation (PolyPhen-2 [49], SIFT [50], Condel [51], CADD [52]), evolutionary conservation in mammals (phastCons [53]) and genomic structure (e.g. segmental duplications) was supplemented using an in-house database.

To identify potentially causal variants, annotated variants were subjected to filtering and prioritization using both functional annotations (e.g. population allele frequency, impact on protein function, conservation etc.) and pedigree information. We performed causal variant prioritization based on four possible modes of inheritance: recessive, compound heterozygous, X-linked and *de novo*. The reduced gene list was furthermore ranked using ENDEAVOUR [54], which has been trained on a list of known genes related to spinocerebellar ataxias (SCA) extracted from Online Mendelian Inheritance in Man (OMIM) [55] and the muscle gene table [56]. The list of training genes consisted of *ABCB7*, *AFG3L2*, *ANO10*, *ATP2B3*, *ATXN1*, *ATXN2*, *ATXN3*, *ATXN7*, *ATXN8*, *ATXN10*, *ATXN8OS*, *BEAN1*, *C10orf2*, *CABC1*, *CACNA1A*, *EEF2*, *FGF14*, *GRM1*, *ITPR1*, *KCNC3*, *KCND3*, *NOP56*, *PDYN*, *PNPLA6*, *POLG*, *PPP2R2B*, *PRKCG*, *SETX*, *SPTBN2*, *SYNE1*, *SYT14*, *TBP*, *TDP1*, *TGM6*, *TPP1*, *TTBK2*, *WWOX*, *ZNF592*. The highest ranking variant in the affected child (*de novo* indel in *CACNA1A*) was selected for further validation and functional studies.

#### Sanger Validation

Direct sequencing was carried out to confirm the *CACNA1A de novo* indel identified through whole-exome sequencing (WES). To that end, *CACNA1A* exon 28 (containing the nucleotide triplet deleted) from the proband and progenitors, was amplified by PCR, purified and sequenced using the BigDye Terminator cycle sequencing kit v3.1 and an automated sequencer ABI PRISM 3730 DNA Analyzer (Applied Biosystems, Foster City, CA, USA) (primer sequences and PCR procedures are available from the authors upon request).

### Ethics Statement

The present investigation was conducted according to the principles expressed in the Declaration of Helsinki and was approved by the Ethics Committee of the Institute for Research in Biomedicine (IRB) at Vall d’Hebron University Hospital. Written informed consent was obtained from the parents for participation in this study, including the genetic analyses in the trio (patient and his parents) and the pathological analyses in the patient.

### cDNA Constructs and Site-Directed Mutagenesis

cDNA of the human voltage-gated Ca^2+^ (Ca_V_2.1) channel α_1A_ subunit (originally cloned into a pCMV vector) was a gift from Professor J. Striessnig (University of Innsbruck, Austria). cDNAs of the rabbit α_2_δ and rat β_3_ regulatory subunits (subcloned into a pcDNA3 expression vector) were gifts from Dr. L. Birnbaumer (National Institutes of Health, North Carolina, USA). Ca_V_2.1 ΔF1502 mutant channel was generated by deleting the CTT codon corresponding to residue F1502 of the human α_1A_ subunit using site-directed mutagenesis (GenScript Corporation, Piscatway, NJ).

All cDNA clones used in this study were sequenced in full to confirm their integrity.

### Heterologous Expression and Electrophysiology

tsA-201 HEK cells were transfected using a linear polyethylenimine (PEI) derivative, the polycation ExGen500 (Fermentas Inc., Hanover, Maryland, USA) as previously reported (eight equivalents PEI/3.3 μg DNA/dish) [13]. Transfection was performed using the ratio for α_1A_ (wild-type (WT) or ΔF1502), β_3_, α_2_δ, and EGFP (transfection marker) cDNA constructs of 1:1:1:0.3. Recordings were done 24–48 h after transfection at room temperature (22–24°C).

Ca^2+^ currents (I_Ca_^2+^) through WT or ΔF1502 Ca_V_2.1 channels were measured using the whole-cell configuration of the patch-clamp technique as described in detail previously [13]. In brief, pipettes had a resistance of 2–3 MΩ when filled with a solution containing (in mM): 140 CsCl, 1 EGTA, 4 Na_2_ATP, 0.1 Na_3_GTP, and 10 HEPES (pH 7.2–7.3 and 290–300 mOsmol/l). The external solution contained (in mM): 140 tetraethylammonium-Cl (TEACl), 3 CsCl, 2.5 CaCl_2_, 1.2 MgCl_2_, 10 HEPES and 10 D-glucose (pH 7.4 and 300–310 mOsmol/l). When measuring Ba^2+^ currents, CaCl_2_ was replaced with an equimolar concentration of BaCl_2_. Recordings were obtained with a D-6100 Darmstadt amplifier (List Medical, Germany) and the pClamp8 software (Axon Instruments, Foster City, CA, USA) was used for pulse generation, data acquisition and subsequent analysis.

Peak inward Ca^2+^ currents in response to 20 ms depolarizing pulses were measured from cells clamped at -80 mV, as described in detail previously [13]. In order to obtain the voltage-dependence of channel activation, single normalized current-voltage (I-V) relationships were fitted with the modified Boltzmann equation, as previously reported [13]:

$$I\;=\;\frac{Gmax(V-Vrev)}{1+e^{\frac{-(V-V1/2\;act)}{kact}}}$$ (1)

where I is the peak current, G_max_ is the maximal conductance of the cell, V is the membrane potential, V_rev_ is the extrapolated reversal potential of I_Ca_^2+^, V_1/2 act_ is the voltage for half-maximal current activation, and k_act_ is the slope factor of the Boltzmann term.

Kinetics of activation, deactivation, inactivation and recovery from inactivation for WT and ΔF1502 Ca_V_2.1 channels were estimated as described in detail previously [13], with slight modifications. In particular, time constants for deactivation (τ_deactivation_) were obtained from single exponential fits of tail currents obtained with 10 ms prepulse to +20 mV (for WT channels) or -5 mV (for ΔF1502 channels) (to maximally open Ca_V_2.1 channels) and followed by test pulses between -80 and 0 mV (in 5 mV steps) for 30 ms; and time course of I_Ca_^2+^ recovery from inactivation was tested by applying a second pulse of 50 ms to +20 mV at increasing time intervals (1–206 s) after an inactivating 3 s prepulse, and relative current at different times (normalized by the peak Ca^2+^ current obtained during the 3s prepulse) was fitted to a single exponential.

The voltage dependence of steady-state inactivation was estimated by measuring peak Ca^2+^ currents in response to depolarizing pulses (to +20 mV for WT channels and to -5 mV for ΔF1502 channels) from a holding of -80 mV, following 30 s steps to various holding potentials (conditioning pulses) between -80 and +5 mV. As described in detail previously [13], half-maximal voltage (V_1/2 inact_) and slope factor (k_inact_) for steady-state inactivation were obtained by fitting normalized I_Ca_^2+^ persistent currents to the following Boltzmann equation:

$$\frac{I}{Imax}\;=\;\frac{1}{1+e^{\frac{V-V1/2\;inact}{kinact}}}$$ (2)

The quality of all fittings performed in the present work was always high, with adjusted R-squared values close to 1 (ranging between 0.95 and 0.99).

Ca^2+^ entry through WT and ΔF1502 Ca_V_2.1 channels was also measured in response to three different action potential-like waveforms (APWs) that have been reported to represent a wide range of action potential durations present in neurons [13,57]. In brief, the duration above half-amplitude and the rising phase slope were, respectively: 0.5 ms and 367 mV/ms for the fast APW, 1 ms and 114 mV/ms for the medium APW and 2.2 ms and 105 mV/ms for the slow APW. All APWs were normalized to a resting potential of -80 mV and peak amplitude of +30 mV). Ca^2+^ currents were acquired at 33 kHz and corrected for leak and capacitive currents using the leak subtraction procedure (P/4). As described previously [13], total Ca^2+^ influx (Q_Ca_^2+^) in response to APWs was calculated as the integral of the Ca^2+^ current delimited by the time point at which inward current initially diverged from the baseline, and the time at which it returned to baseline. That time was considered as the time for Ca^2+^ entry. Trains of 1000 fast and medium APWs (applied at the highest frequency allowed by the corresponding electrophysiological protocol including P/4 leak subtraction procedure: 50 Hz and 42 Hz, respectively) were applied in some experiments in order to measure Ca^2+^ entry through WT and ΔF1502 Ca_V_2.1 channels in response to repetitive, brief depolarization of physiological relevance in neurons. Accumulative Ca^2+^ influx along the different trains of APWs was calculated by adding the Q_Ca_^2+^ values obtained for each single APW stimulus in the corresponding train.

### Statistics

Data are presented as the means ± S.E.M. Statistical tests included unpaired or paired Student’s *t* test or, Mann-Whitney U-test, as appropriate. Differences were considered significant if P < 0.05.

## Results

### ΔF1502 Ca_V_2.1 Mutant in a Spanish Case of Congenital Ataxia

This 7 year-old boy was normally born after an uneventful pregnancy to unrelated parents. He was noted to be hypotonic and lag behind in motor development over the first months of life. A younger 3 year-old brother is healthy. Examination at the age of 5 months, revealed an alert, non-dysmorphic infant, with good visual fixation but saccadization of pursuit gaze; there was global hypotonia without any evidence of muscle weakness or atrophy; deep tendon reflexes were preserved. Motor development was delayed from early on: he attained head control by age 6 months, sat unassisted at age 3 years. Currently remains in the upright position with aid of a stander and uses a wheel chair for ambulation.

**Fig 1:**
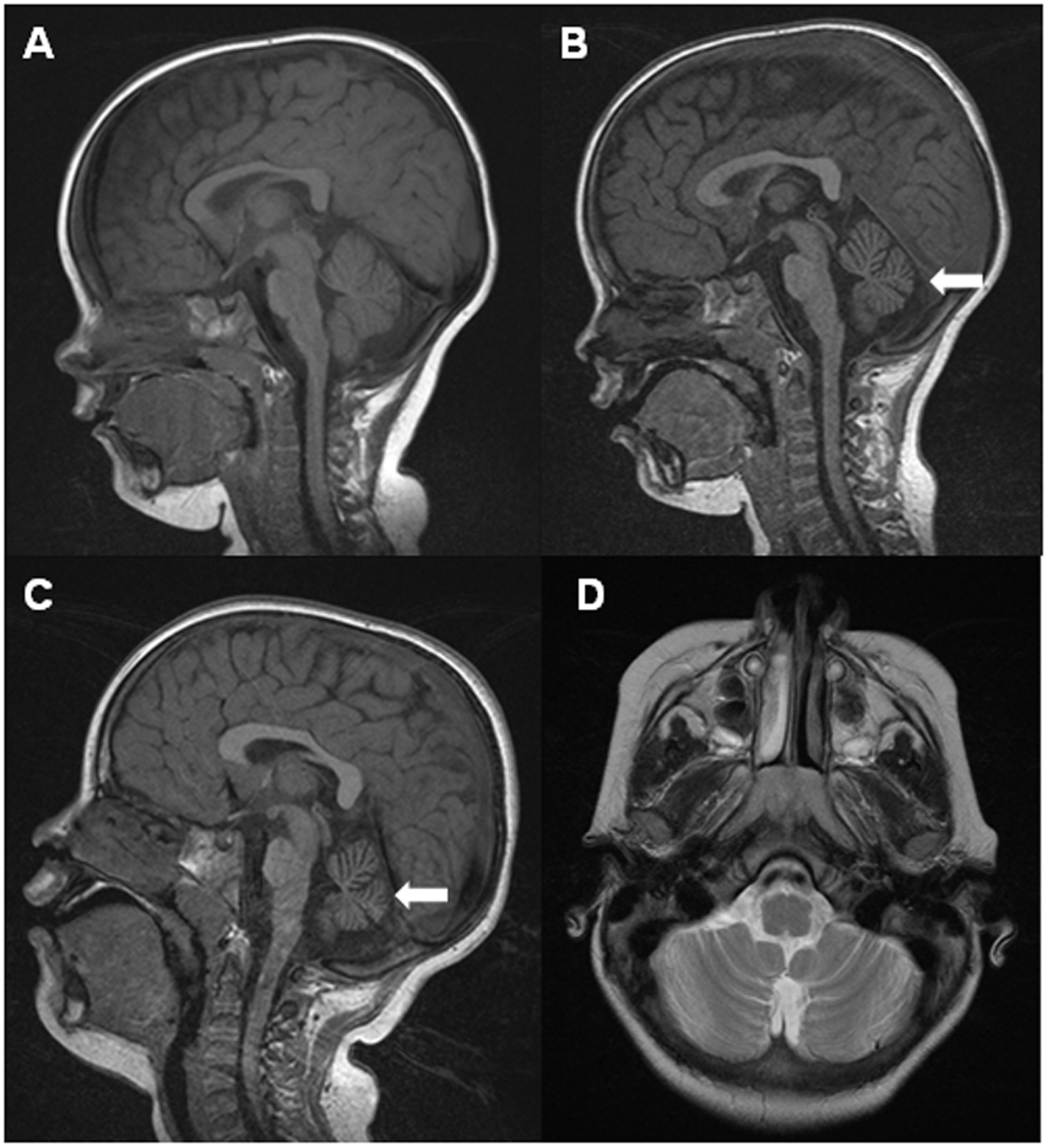
Brain MRI of the proband at the age of 14 months (A), 28 months (B), and 4 and a half years (C,D). After the initial normal findings (A), note the progressive cerebellar atrophy mainly involving the complete vermis (indicated by the arrows in B, C). The hemispheres, displaying prominence of the cerebellar folia, were eventually affected (D).

At the age of 7 years the patient has developed a complete ataxic syndrome. He is able to understand simple commands and speaks in short sentences. He is quite sociable and attends a special school. He is able to draw or self-feed with spoon. On examination, he is normocephalic and there are no telangiectasias or organomegalies. There is a fine, conjugate horizontal/rotary nystagmus and a complex alteration of ocular pursuit that resembles oculomotor apraxia, prominent head and trunk titubation, diffuse hypotonia, normal strength, brisk deep-tendon reflexes, flexor plantar responses and mild upper limb dysmetria with no tremor. He can adopt a quadrupedal position but is not able to stand or walk holding onto furniture. Ancillary tests included serum creatine kinase, vitamin E, alpha fetoprotein, immunoglobulins, thyroid hormones, amino acids, transferrin isoelectric focusing, urine organic acids, CSF lactate, pterins, folate and dopamine and serotonin metabolites. A comparative genomic hybridization array showed a 225 Kb segmental monosomy in the sexual chromosomes pseudoautosomal region (PAR2). Although this rearrangement affected the dose of *SPRY3* and *VAMP7*, it was also found in his asymptomatic father. Fibroblast beta-galactosidase, beta-D-glucuronidase, beta-glucosidase, beta-N-acetyl-glucosaminidase, hexosaminidase A, alpha-galactosidase and alpha-glucosidase activities were normal. A neurophysiological study showed normality of motor and sensory neurography, EMG and somatosensory evoked responses. Visual evoked potentials (flash) and electroretinogram were normal. A muscle biopsy disclosed normal histochemistry and normal activities of the respiratory chain enzymes. A brain magnetic resonance imaging (MRI) at the age of 14 months did not reveal alterations. Subsequent studies performed at the ages of 28 months and 4 years showed a conspicuous and progressive, predominantly vermian, cerebellar atrophy with no involvement of other brain areas (Fig 1).

**Fig 2:**
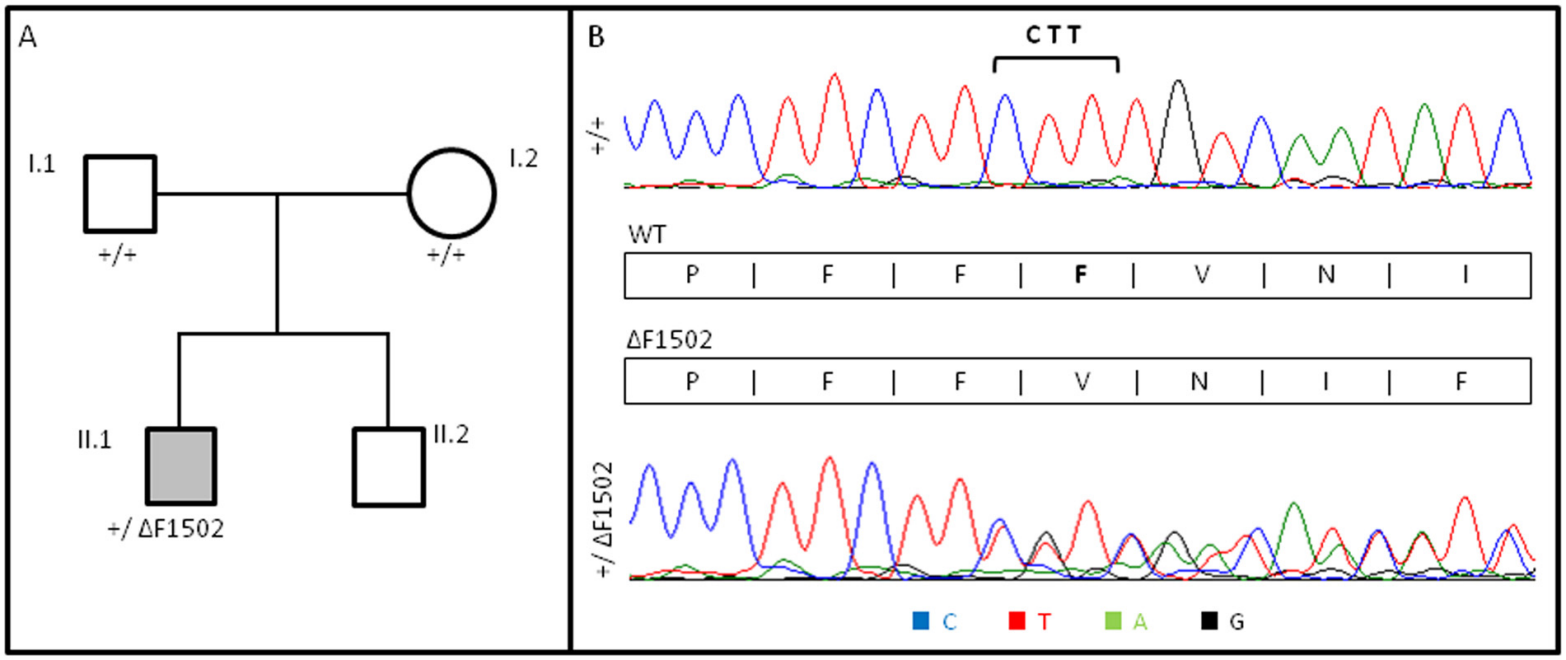
*De novo* heterozygous *CACNA1A* deletion in congenital ataxia with cerebellar atrophy. (A) Pedigree of the affected individual carrying the *de novo* heterozygous ΔF1502 mutation. White symbols denote healthy individuals and grey, congenital ataxia. (B) Electropherograms showing the deleted nucleotides (bracket) (NM_001127221.1-transcript variant 3:c.4503-4505delCTT) leading to a F1052 deletion (NP_001120693.1). Note the double wild-type (WT) and mutant (ΔF1502) sequence in the patient’s electropherogram (heterozygous mutation carrier).

Due to the large number of genes potentially causing cerebellar ataxia, we performed whole exome sequencing (WES) of a parent-child trio with an unknown cause of congenital ataxia in the child. Variant detection on the sequencing data using GATK [45] and ClinDel [47] yielded 115,818 and 12,753 variants, respectively. Variants affecting the protein sequence (nonsynonymous or splicing) were filtered based on possible inheritance modes, including *de novo*, recessive, compound heterozygous or X-linked. Variants coherent with one of the inheritance modes were further prioritized based on population allele frequency in EVS and 1000GP (i.e. rare variants with < 1% AF), damage potential based on PolyPhen-2, SIFT, Condel and CADD [49–52], evolutionary conservation based on phastCons [53] and finally sequence complexity and repetitiveness (e.g. variants in segmental duplications are removed), finally yielding a total of seven *de novo*, eight recessive and 15 compound heterozygous variants that are rare or novel and highly damaging. The affected 30 genes were subsequently subjected to additional prioritization using ENDEAVOUR [54], which has been trained on genes extracted from various disease gene resources [55,56]. The ENDEAVOUR prioritization highlighted a *de novo* triplet deletion in *CACNA1A* (NM_001127221.1-transcript variant 3:c.4503-4505delCTT) as most likely candidate, as being part of the list of training genes. Furthermore, no other genes after filtering could be linked to the investigated phenotype. Manual review of the respective alignment data supports the presence of the deletion that was confirmed as a *de novo* variant by Sanger sequencing (Fig 2). This heterozygous *CACNA1A* deletion brings about a ΔF1502 change in the Ca_V_2.1 α_1A_ channel subunit, previously described in association with both congenital ataxia and hemiplegic migraine [44].

### ΔF1502 Alters Current Density and Activation, Deactivation and Inactivation Features of Human Ca_V_2.1 Channels

**Fig 3:**
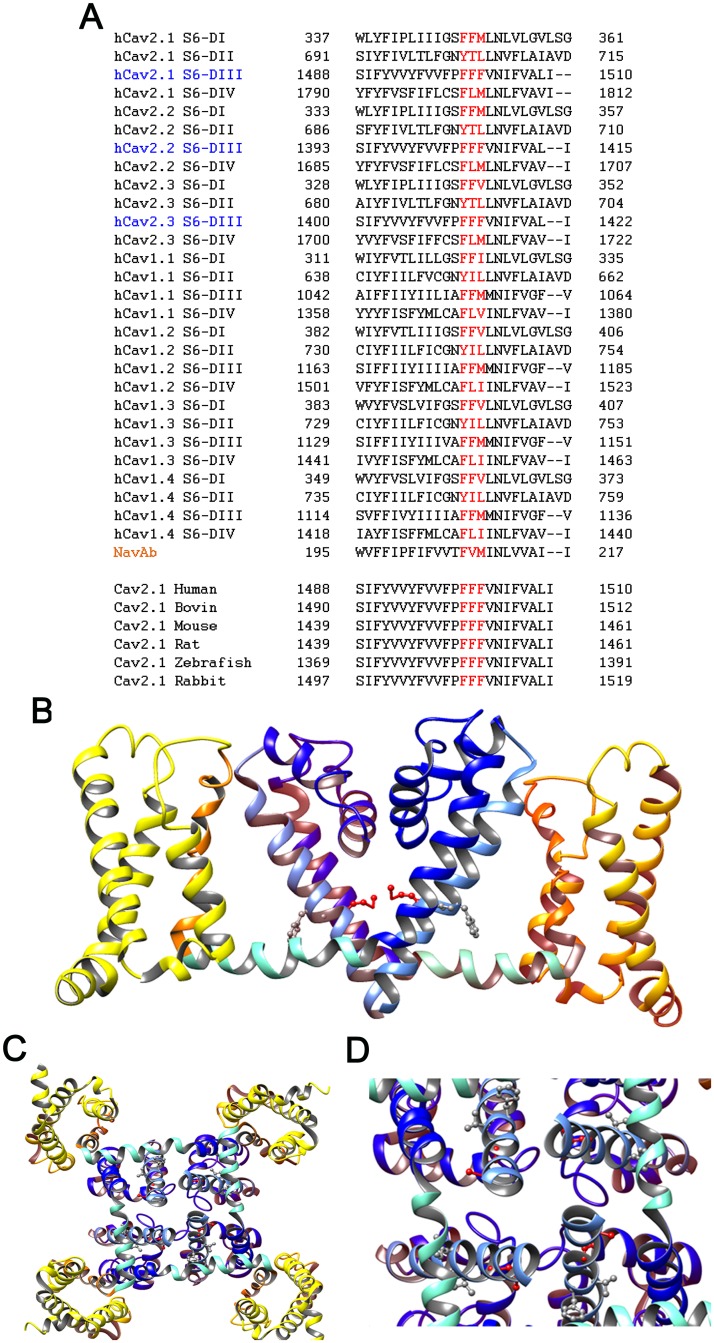
Evolutionary conservation of the F1502 residue and predicted location at the channel pore. (A) Sequence alignment of individual S6 segments at domains I to IV (DI-DIV) of human Ca_V_2.x channel α_1_ subunits (P/Q type Ca_V_2.1; N-type Ca_V_2.2; R-type Ca_V_2.3), human Ca_V_1.x (L-type) channel α_1_ subunits, and the bacterial sodium channel Na_V_Ab (top); sequence alignment of S6-DIII of Ca_V_2.1 channels from different species (as indicated). The three Phenylalanine’s group (in red) is conserved in the human Ca_V_2.1 channel α_1A_ subunit, where F1502 is located at the third position. This particular amino acid residue is only conserved in S6-DIII of Ca_V_2 type channels. The phenylalanine’s group is totally conserved in S6-DIII of Ca_V_2.1 channels from different species. The alignments were performed with T-Coffee (T-Coffee). (B,C,D) Location of the F1502 homologous methionine residue (M209), using the Na_V_Ab structure as a model (PDB 4EKW). A methionine residue is also present at the F1502 position in L-type channels. The side view (B) show a red highlighted M209 residue in Na_V_Ab, which lines the inner pore vestibule of the channel. A view from the cytoplasm looking up through the channel pore show the arrangement of M209 residue in the four Na_V_Ab subunits (C), and a zoom of the pore region from the same view is shown in (D). Images were generated using UCSF Chimera package. Chimera is developed by the Resource for Biocomputing, Visualization, and Informatics at the University of California, San Francisco (supported by NIGMS P41-GM103311) [79].

The F1502 residue of the human Ca_V_2.1 α_1A_ channel subunit is the third amino acid containing a non-polar aromatic side chain of the three phenylalanine’s group (F1500, F1501, F1502), highly conserved in the pore-forming region of the channel. Thus, F1502 residue lies in all S6 segments of domains III (DIII-S6) of all members of the human Ca_V_2 channel family (Ca_V_2.1 or P/Q-type, Ca_V_2.2 or N-type and Ca_V_2.3 or R-type channels). Besides, F1502 is also conserved through evolution as shown by a comparison of orthologous Ca_V_2.1 channels (Fig 3A). In human Ca_V_1.x and bacterial voltage-gated Na^+^ (Na_V_Ab) channels, a different non-polar but aliphatic side chain amino acid (methionine) occupy the equivalent F1502 residue position (Fig 3B). Ca_V_2.1 F1502 is located eight residues upstream of a highly conserved isoleucine contributing to the ring of hydrophobic residues that seems to form the internal pore gate of voltage-gated ion channels [58]. F1502 homologous methionine residue in the Na_V_Ab crystal structure is facing to the channel pore, without any evident interaction with other segments either in the pore or the voltage-sensing regions (Fig 3B, 3C and 3D).

**Fig 4:**
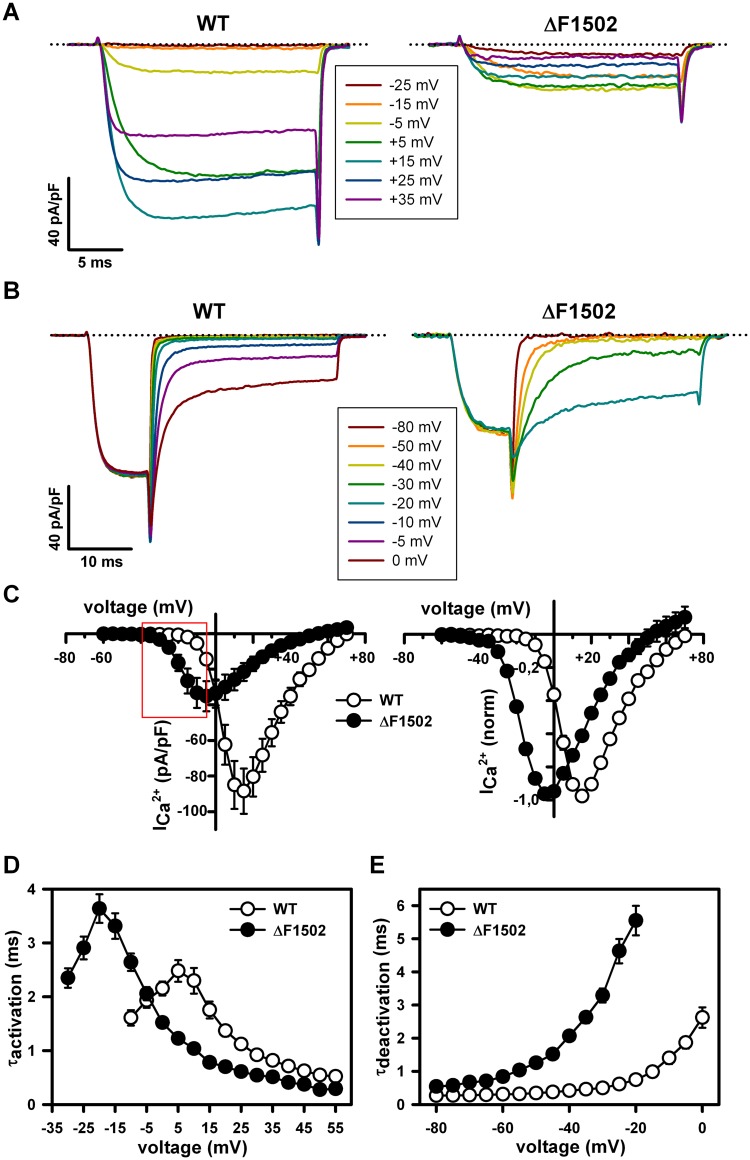
ΔF1502 induces a gain-of-function in the heterologously expressed Ca_V_2.1 channel by affecting its activation and deactivation properties. (A) Representative current traces elicited by 20 ms depolarizing pulses from -80 mV to the indicated voltages (inset), illustrating the difference in voltage-dependence and activation kinetics between wild-type (WT) (left) and ΔF1502 (right) Ca_V_2.1 channels. Dotted lines indicate the zero current level. (B) Representative current traces showing distinct deactivation kinetics of WT (left) and ΔF1502 (right) Ca_V_2.1 channels, obtained by hyperpolarizing the cells during 30 ms at the indicated voltages (inset) following a 20 ms depolarizing pulse to +20 mV (for WT channels) or -5 mV (for ΔF1502 channels). The zero current level is indicated by dotted lines. (C) Average current density-voltage relationships (left) and normalized I-V curves (right) for WT (open circles, n = 27) and ΔF1502 (filled circles, n = 19) Ca_V_2.1 channels expressed in tsA-201 HEK cells. Red box indicates the voltage range at which peak Ca^2+^ current densities through ΔF1502 channels exceed those produced by WT channels. Average V_1/2 act_, k_act_ and V_rev_ values were (in mV): WT (open circles, n = 27) 3.8 ± 0.6, 3.5 ± 0.15 and 62.4 ± 1.4; ΔF1502 (filled circles, n = 19) -17.1 ± 0.9, 4.4 ± 0.19 and 51.6 ± 2.2, respectively. Average activation (D) and deactivation (E) kinetics of WT (open circles) and ΔF1502 Ca_V_2.1 Ca^2+^ currents (filled circles) at the indicated voltages.

Maximal Ca^2+^ current densities for expressed mutant ΔF1502 α_1A_ in tsA-201 HEK cells were ~ 60% smaller than current densities for wild-type (WT) α_1A_ channels (Fig 4A and 4C, left panel). The potential for half-maximal activation (V_1/2 act_) was strongly left-shifted for ΔF1502 Ca_V_2.1 channels by ~ 21 mV (P < 0.0001, Student’s *t* test), with a significant 0.9 mV increase (P < 0.001, Student’s *t* test) in the steepness of the activation curve (k_act_) (Fig 4C, right panel; mean ± SEM values for V_1/2 act_ and k_act_ are provided in the figure legend). Accordingly, the maximal Ca^2+^ current amplitude was induced by depolarizing pulses to +15 mV or -5 mV for WT or ΔF1502 channels, respectively (Fig 4C). Due to this ΔF1502-induced left-shift in Ca_V_2.1 channel activation, Ca^2+^ current densities through mutant channels were significantly higher than current densities through WT channels (P < 0.05–0.0001, Mann-Whitney U-test) in a range of depolarized voltages with physiological relevance in neurons (from -40 to -5 mV) (Fig 4C, left panel; S1 Table). Activation and deactivation kinetics were also considerably left-shifted in the ΔF1502 channel (Fig 4D and 4E, respectively): the highest *τ*_activation_ for WT (2.5 ± 0.2 ms, n = 27) or ΔF1502 (3.4 ± 0.3 ms, n = 19) Ca^2+^ currents were observed at +5 mV and -20 mV, respectively. In addition, activation kinetics of ΔF1502 channels were significantly accelerated (P < 0.05–0.0001, Mann-Whitney U-test) in a wide range of depolarizing voltages (from 0 to +55 mV), when compared to WT channels (Fig 4A and 4D; S2 Table). Concerning gate closing, ΔF1502 Ca_V_2.1 channels presented slower *τ*_deactivation_ at voltages from -80 to -20 mV (Fig 4B and 4E; S3 Table, P < 0.0001, Mann-Whitney U-test).

**Fig 5:**
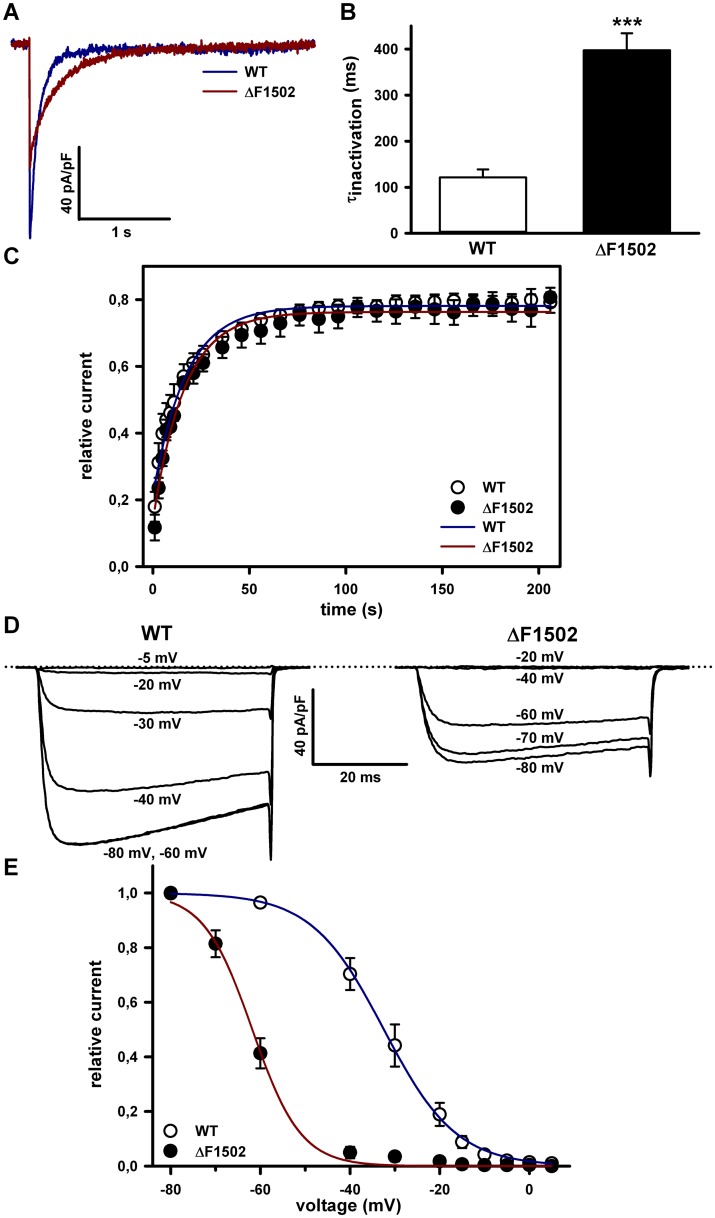
ΔF1502 affects Ca_V_2.1 channel inactivation properties. (A) Representative current traces illustrating the slower inactivation kinetics for ΔF1502 Ca_V_2.1 channels (red trace) when compared to WT channels (blue trace), in response to a 3 s depolarizing pulse to +20 mV. (B) Average τ_inactivation_ values of Ca^2+^ currents through WT (open bar, n = 10) and ΔF1502 (filled bar, n = 8) Ca_V_2.1 channels, elicited as indicated in panel (A). (C) Similar time course of Ca^2+^ current recovery from inactivation for WT and ΔF1502 Ca_V_2.1 channels. Average τ of current recovery from inactivation obtained after fitting the data to a single exponential (solid color lines), were (in s): WT (open circles, n = 7) 15.5 ± 1.1; ΔF1502 (filled circles, n = 5) 16.9 ± 2.1 (P = 0.5, Student’s *t* test). (D, E) Steady-state inactivation of WT and ΔF1502 Ca_V_2.1 channels. Amplitudes of currents elicited by test pulses to +20 mV (for WT channels) or -5 mV (for ΔF1502 channels) were normalized to the current obtained after a 30 s prepulse to -80 mV and fitted by a single Boltzmann function (solid color traces) (see Materials and Methods, Eq 2). Average V_1/2 inact_ and k_inact_ values were (in mV): WT (open circles, n = 19) -32.2 ± 2.1 and -5.3 ± 0.3; ΔF1502 (filled circles, n = 12) -60.7 ± 1 and -5.2 ± 0.8, respectively. No significant difference was found for k_inact_ values (P = 0.89, Mann-Whitney test).

Next, we studied whether ΔF1502 affects the time course of channel inactivation by analyzing the Ca^2+^ current decay during a 3-s test pulse elicited from a holding potential of -80 mV to +20 mV (Fig 5A). We found that inactivation kinetic for ΔF1502 Ca_V_2.1 Ca^2+^ currents was significantly slower (τ_inactivation_ = 397.3 ± 37.1 ms, n = 8) than for WT currents (τ_inactivation_ = 121.3 ± 17.4 ms, n = 10) (Fig 5B, P < 0.0001, Student’s *t* test). However, the rate of recovery from inactivation was unaffected in ΔF1502 channels (Fig 5C, P = 0.5, Student’s *t* test). The half-maximal voltage for steady-state inactivation (V_1/2 inact_) induced by 30s conditioning prepulses between -80 and +5 mV was greatly left-shifted (~ 28.5 mV) in ΔF1502 channels (P < 0.0001, Student’s *t* test), without significant change in the steepness of the inactivation curve (symbolized by k_inact_) (P = 0.89, Mann-Whitney test) (Fig 5D and 5E).

### Effect of ΔF1502 over Ca_V_2.1 Mediated Ca^2+^ Influx Evoked Either by Single or Trains of Action Potential-Like Waveforms

**Fig 6:**
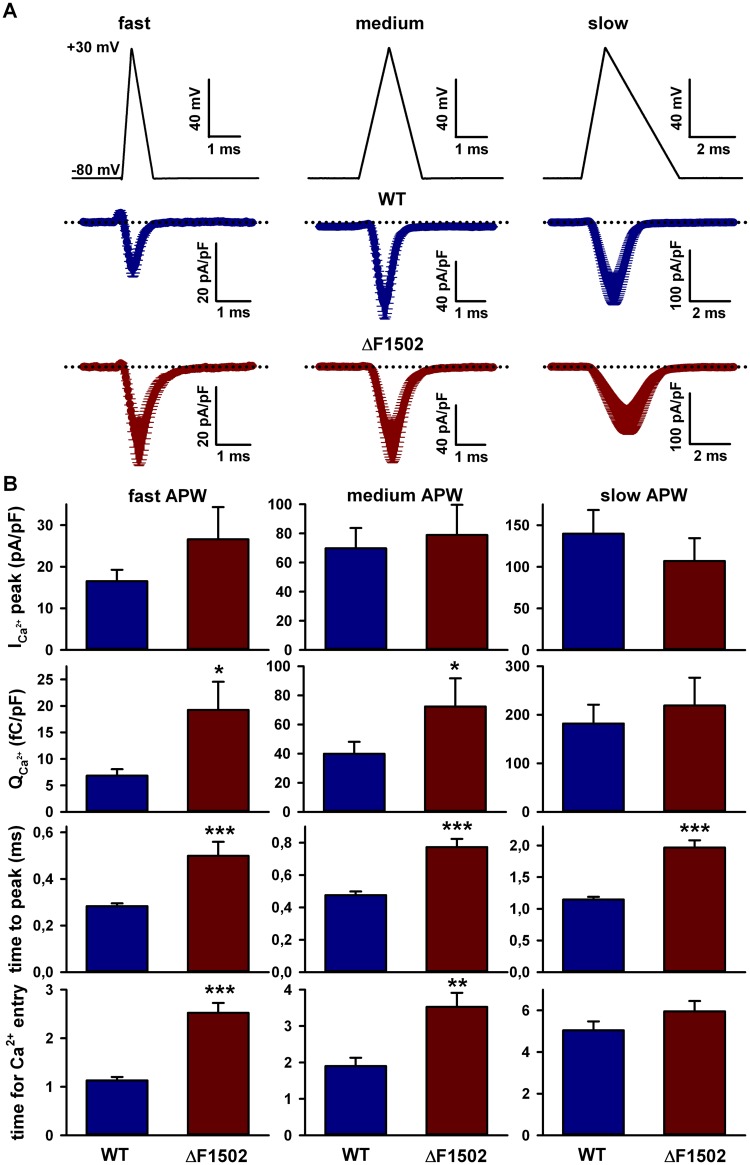
ΔF1502 effects on Ca^2+^ influx evoked by single action potential-like waveforms (APWs). (A) Average Ca^2+^ current traces evoked by APWs of different durations (fast (left panels), medium (central panels) and slow (right panels) (see Materials and Methods for details) obtained from tsA-201 HEK cells expressing WT (blue traces) or ΔF1502 (red traces) Ca_V_2.1 channels. Dotted lines stand for the zero current level. (B) Average data for peak Ca^2+^ (I_Ca_^2+^) current density (top panel), normalized Ca^2+^ influx (Q_Ca_^2+^) (second panel), time to peak (third panel), and time for Ca^2+^ entry (bottom panel) in response to APWs of different durations obtained from cells expressing WT (blue bars, n = 12–14) or ΔF1502 (red bars, n = 8) Ca_V_2.1 channels (*P < 0.05, **P < 0.001 and ***P < 0.0001 when compared to WT).

To better understand the physiological impact of ΔF1502 effects on Ca_V_2.1 channel activity, we measured the Ca^2+^ influx through WT and mutant channels elicited by single action potential-like waveforms (APWs) of different durations (fast, medium and slow; Fig 6A, top; for further details see Materials and Methods [13,57]). In spite of the significant reduction in maximal Ca^2+^ current density produced by ΔF1502 (in this set of experiments maximal Ca^2+^ current density through ΔF1502 channels in response to a 20 ms depolarizing pulse was ~ 50% smaller when compared to WT current, S1 Fig, left panel), no significant changes among cells expressing either WT or ΔF1502 channels were observed regarding the peak Ca^2+^ current density elicited by the different APWs (Fig 6A and 6B, top panel). Besides, and more important, total Ca^2+^ influx (Q_Ca_^2+^) in response to APWs was not reduced by ΔF1502. Not only that, but the amount of Ca^2+^ that entered into the cell in response to fast and medium APWs was significantly higher for cells expressing the Ca_V_2.1 mutant channel (Fig 6A and 6B, second panel), most probably due to the ~ 18.7 mV ΔF1502-induced left-shit in the activation curve observed in this set of cells (S1 Fig, right panel). This, in combination with the effects that ΔF1502 exerts on channel activation and deactivation kinetics, may also explain another two facts: the time required to reach the peak current density in response to all APWs was always significantly higher in cells expressing the mutant Ca_V_2.1 channel (Fig 6A and 6B, third panel); and the time of Ca^2+^ entry in response to fast and medium APWs was significantly higher in cells expressing the mutant Ca_V_2.1 channel (Fig 6A and 6B, bottom panel).

**Fig 7:**
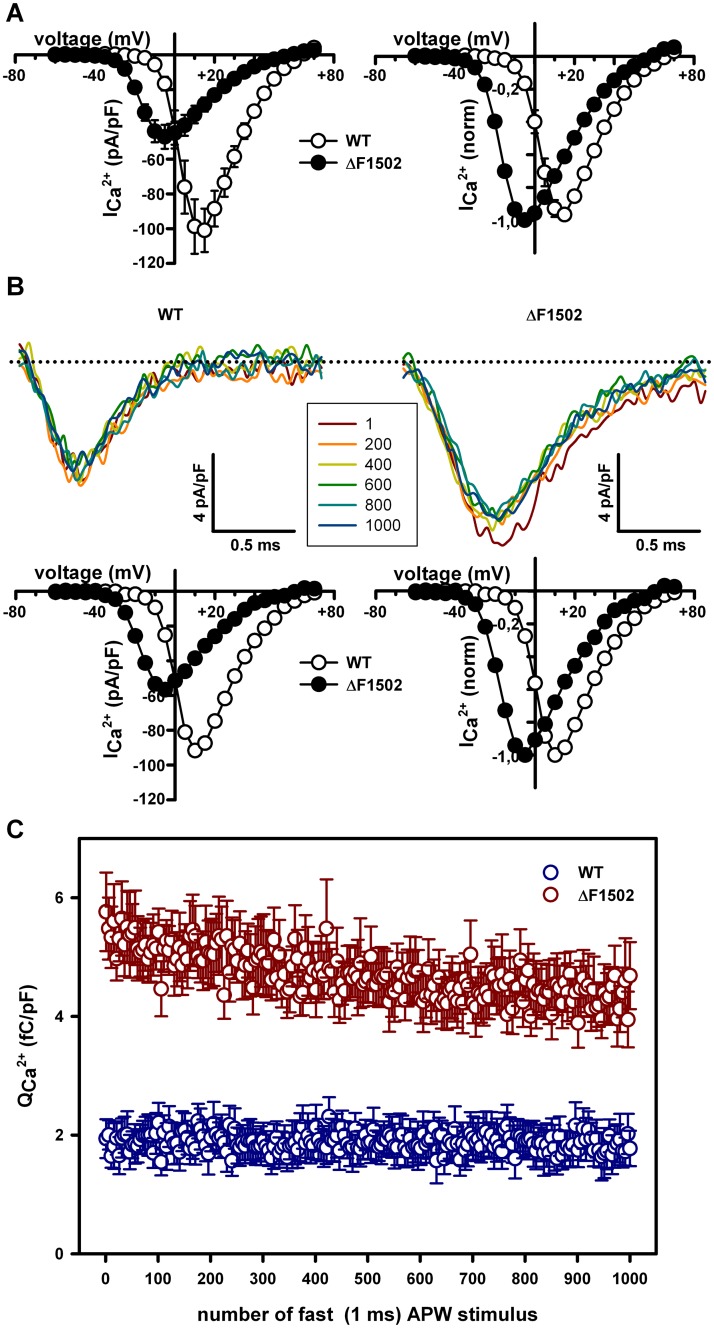
ΔF1502 effects on Ca^2+^ influx evoked by a 50 Hz train of 1 ms action potential-like waveforms (APWs). (A) Average current density-voltage relationships (left) and normalized I-V curves (right) for WT (open circles, n = 9) and ΔF1502 (filled circles, n = 7) Ca_V_2.1 channels expressed in tsA-201 HEK cells, before stimulation with a 50 Hz train of 1 ms APWs. In this series of experiments, maximal Ca^2+^ current density through Ca_V_2.1 channels is still significantly reduced by ΔF1502 (left panel: from -100.92 ± 12.5 pA/pF (for WT, n = 9) to -47.18 ± 6.9 pA/pF (for ΔF1502, n = 7), P < 0.01, Student’s *t* test) and the significant left-shift induced by ΔF1502 on the Ca_V_2.1 voltage-dependent activation is also present (right panel: WT V_1/2 act_ = 2.27 ± 1.3 mV (n = 9) *versus* ΔF1502 V_1/2 act_ = -17.73 ± 0.4 mV (n = 7), P < 0.0001, Student’s *t* test). (B) Representative Ca^2+^ current traces evoked by every 200^th^ pulse of a 50 Hz train of fast (1 ms) APWs (see Materials and Methods for details) obtained from two tsA-201 HEK cells expressing either WT (left) or ΔF1502 (right) Ca_V_2.1 channels. Dotted lines stand for the zero current level. The corresponding current density-voltage relationships (left) and normalized I-V curves (right), obtained from these two cells before stimulation with a 50 Hz train of fast APWs, are shown at the bottom (maximal Ca^2+^ current density through WT and ΔF1502 Ca_V_2.1 channels are -91.7 pA/pF and -56.7 pA/pF, respectively; V_1/2 act_ values for WT and ΔF1502 Ca_V_2.1 channels are -0.5 mV and -18.97 mV, respectively). (C) Average data for Ca^2+^ influx normalized by cell size (Q_Ca_^2+^) in response to every 5^th^ pulse of a 50 Hz train of fast (1 ms) APWs, obtained from cells expressing WT (blue symbols, n = 9) or ΔF1502 (red symbols, n = 7) Ca_V_2.1 channels.

**Fig 8:**
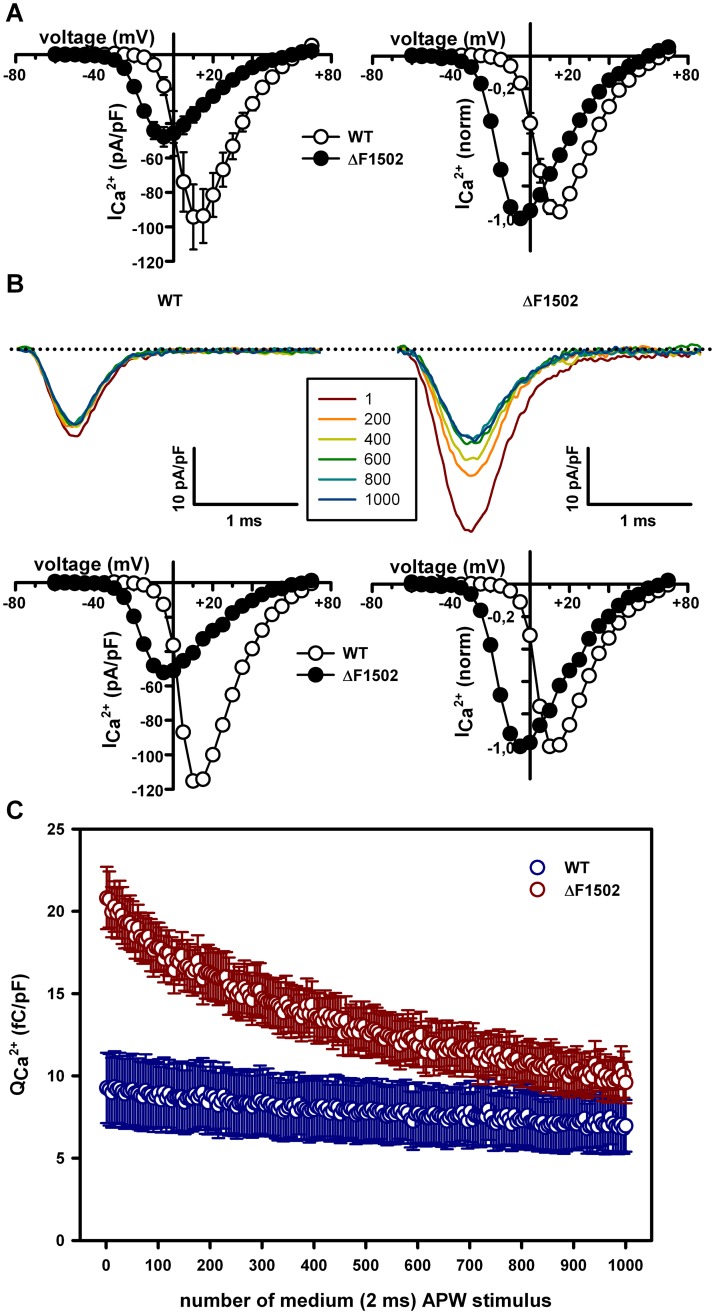
ΔF1502 effects on Ca^2+^ influx evoked by a 42 Hz train of 2 ms action potential-like waveforms (APWs). (A) Average current density-voltage relationships (left) and normalized I-V curves (right) for WT (open circles, n = 10) and ΔF1502 (filled circles, n = 11) Ca_V_2.1 channels expressed in tsA-201 HEK cells, before stimulation with a 42 Hz train of 2 ms APWs. In this series of experiments, maximal Ca^2+^ current density through Ca_V_2.1 channels is still significantly reduced by ΔF1502 (left panel: from -94.26 ± 18.9 pA/pF (for WT, n = 10) to -47.76 ± 5.7 pA/pF (for ΔF1502, n = 11), P < 0.05, Student’s *t* test) and the significant left-shift induced by ΔF1502 on the Ca_V_2.1 voltage-dependent activation is also noticed (right panel: WT V_1/2 act_ = 2.32 ± 1.18 mV (n = 10) *versus* ΔF1502 V_1/2 act_ = -17.74 ± 0.35 mV (n = 11), P < 0.0001, Student’s *t* test). (B) Representative Ca^2+^ current traces evoked by every 200^th^ pulse of a 42 Hz train of medium (2 ms) APWs (see Materials and Methods for details) obtained from two tsA-201 HEK cells expressing either WT (left) or ΔF1502 (right) Ca_V_2.1 channels. Dotted lines stand for the zero current level. The corresponding current density-voltage relationships (left) and normalized I-V curves (right), obtained from these two cells before stimulation with a 42 Hz train of 2 ms APWs, are shown at the bottom (maximal Ca^2+^ current density through WT and ΔF1502 Ca_V_2.1 channels are -115.28 pA/pF and -52.27 pA/pF, respectively; V_1/2 act_ values for WT and ΔF1502 Ca_V_2.1 channels are 2.52 mV and -17.23 mV, respectively). (C) Average data for Ca^2+^ influx normalized by cell size (Q_Ca_^2+^) in response to every 5^th^ pulse of a 42 Hz train of medium (2 ms) APWs, obtained from cells expressing WT (blue symbols, n = 10) or ΔF1502 (red symbols, n = 11) Ca_V_2.1 channels.

As ΔF1502 strongly promotes Ca_V_2.1 channel steady-state inactivation by voltage in response to prolonged depolarization (Fig 5D and 5E), we also evaluated if this last action is relevant for Ca^2+^ influx through the channel under physiological conditions. *In vivo*, neurons do not experience long depolarization to induce Ca_V_2.1 steady-state inactivation. Instead, this effect on Ca_V_2.1 channel activity is mimicked by the occurrence of more physiological stimuli, such as trains of action potentials causing repetitive, brief depolarization [59]. Therefore, given that total Ca^2+^ influx (Q_Ca_^2+^) in response to single fast and medium APWs was favored by ΔF1502, we studied the effect of the mutation on Q_Ca_^2+^ elicited by trains of fast and medium APWs applied at relatively high frequency in two new sets of experiments, where it can be observed again the reduction in maximal Ca^2+^ current density (by ~ 50%) and the shift of the current activation curve to lower voltages (~ 20 mV) induced by ΔF1502 (Figs 7A and 8A). The application of a train of fast APWs (1 ms duration, with maximal depolarization to +30 mV from a holding potential of -80 mV, applied at 50 Hz for 20 seconds) did not cause a significant reduction in Ca^2+^ influx through WT Ca_V_2.1 channels (Q_Ca_^2+^ produced by the first APW applied was 1.94 ± 0.3 fC/pF (n = 9) and at the end of the train the observed Q_Ca_^2+^ value was 1.76 ± 0.3 fC/pF (n = 9), P = 0.23, paired Student’s *t* test). However, and according to the effect of ΔF1502 on Ca_V_2.1 channel steady-state inactivation, the same train of fast APWs produced a small, but significant, reduction (by ~ 19%) in Ca^2+^ influx through ΔF1502 Ca_V_2.1 channels (from 5.76 ± 0.7 fC/pF to 4.69 ± 0.6 fC/pF (n = 7), P < 0.01, paired Student’s *t* test) (Fig 7B and 7C). Nevertheless, in spite of this Ca^2+^ entry reduction along the train produced by ΔF1502, Q_Ca_^2+^ after each APW stimulus was always higher for mutant channels (Fig 7B and 7C) and, therefore, accumulative Ca^2+^ influx through ΔF1502 Ca_V_2.1 channels was still significantly higher than through WT channels (4.7 ± 0.4 pC/pF (n = 7) *versus* 1.9 ± 0.3 pC/pF (n = 9), P < 0.0001, Student’s *t* test). Similar conclusions can be drawn from the analysis of studies using trains of medium APWs (2 ms duration, with maximal depolarization to +30 mV from a holding potential of -80 mV, applied at 42 Hz for 23.81 seconds) (Fig 8). In this case, the train of APWs produced a significant reduction in Ca^2+^ influx through both, WT (from 9.27 ± 2.14 fC/pF to 6.97 ± 1.57 fC/pF (n = 10), P < 0.01, paired Student’s *t* test) and ΔF1502 (from 20.81 ± 1.9 fC/pF to 9.6 ± 1.3 fC/pF (n = 11), P < 0.0001, paired Student’s *t* test) Ca_V_2.1 channels, which was significantly greater for mutant channels (54.65 ± 4.4% (n = 10) *versus* 23.69 ± 2.9% (n = 11), P < 0.0001, Student’s *t* test) (Fig 8B and 8C). Yet, accumulative Ca^2+^ influx through ΔF1502 Ca_V_2.1 channels all along the train was significantly higher than through WT channels (13.4 ± 1.4 pC/pF (n = 11) *versus* 7.9 ± 1.8 pC/pF (n = 10), P < 0.05, Student’s *t* test) (Fig 8B and 8C).

## Discussion

Genetic variants in the gene encoding the pore-forming Ca_V_2.1 (P/Q-type) channel α_1A_ subunit (*CACNA1A*) result in heterogeneous human neurological disorders of autosomal dominant inheritance, including familial and sporadic hemiplegic migraine (FHM/SHM), episodic ataxia type 2 (EA2), and spinocerebellar ataxia type 6 (SCA6). Although hemiplegic migraine (HM) is linked to *CACNA1A* mutations that enhance Ca_V_2.1 channel activity and EA2, on the contrary, is associated to loss-of-function mutations, symptomatology overlap of HM, EA2 and SCA6 are well recognized at the clinical level (reviewed in [11]). As yet, it is not clear why some *CACNA1A* mutations cause pure FHM and other FHM with cerebellar signs. Functional studies *in vitro* show different effects on Ca_V_2.1 steady-state inactivation, which can be increased, decreased, or unaltered depending on the mutation [11]. Besides, although all studied mutations share a consistent gain-of-function effect on Ca_V_2.1 channel activation, there are no noticeable differences among these two groups of FHM mutations, with the exception of mutations D715E, S218L [11,18,60–62] and now ΔF1502 (this report). Hence, pure human FHM mutations such as R192Q and Y1245C (within voltage sensor domains of Ca_V_2.1 α_1A_ subunit) or V714A (affecting the pore S6 segment of α_1A_ domain II) (S2 Fig) shift Ca_V_2.1 channel activation to hyperpolarizing voltages by ~ 9 mV [13,16,17,63]. Likewise, human FHM mutations T501M and R583Q (located at voltage sensor domains, S2 Fig), which are linked to progressive cerebellar ataxia (with cerebellar atrophy in the case of R583Q) [14], decrease voltage-threshold for channel activation by ~ 7–10 mV [14,60]. The same applies to human FHM mutations T666M, I1811L (within pore domains) and R1350Q (also referred as R1349Q and homologous to the mouse R1252Q allele of the tottering, *Cacna1a* ^*tg*^, mutant series; located at the voltage sensor S4 segment of α_1A_ domain III) (S2 Fig), which are linked to congenital ataxia [38–43] and shift Ca_V_2.1 channel activation to lower voltages by ~ 6–12 mV [63,64]. However, mutation D715E (located at the pore S6 segment of α_1A_ domain II, S2 Fig) induces a bigger decrease in the voltage-threshold for Ca_V_2.1 channel activation (by ~ 17 mV) [60]. Likewise, the genetic variant S218L (at the loop between S4 and S5 segments of α_1A_ domain I, S2 Fig) displays one of the largest gains of function on channel activation when compared with other FHM mutations, promoting Ca^2+^ influx even at small depolarization, which are insufficient to open wild-type (WT) channels. Such effect can be further potentiated by the slower inactivation kinetics and increased rate of recovery from inactivation reported for the S218L Ca_V_2.1 channel [18,62]. These distinctive effects of mutations D715E and S218L may be enough to explain the severity of the cerebellar symptoms accompanying FHM in the carrier patients, and in particular the more severe clinical phenotype associated to S218L that, in addition to HM attacks and slowly progressive cerebellar ataxia and atrophy, also includes epileptic seizures, coma or profound stupor, and, sometimes fatal, cerebral edema [36,37,39]. Recently, García-Segarra et al. have reported the deletion of the highly conserved phenylalanine 1502 (ΔF1502, located at the S6 pore region of α_1A_ domain III), in association to a case of HM and congenital ataxia [44]. Their study about the functional consequences of ΔF1502 on Ca_V_2.1 channel function in *Xenopus* oocytes using Ba^2+^ as the charge carrier (instead of the physiological permeant ion Ca^2+^), revealed: 1) an ~ 11 mV hyperpolarizing shift in Ca_V_2.1 voltage-dependent activation, 2) a 30% decrease in Ba^2+^ current density at high depolarizing voltages, 3) a shift in the voltage dependence of steady-state inactivation to hyperpolarizing voltages by ~ 14 mV, and 4) a slightly slower recovery from channel inactivation. Although the gain-of-function effect on channel activation fits well with a pathophysiological role of mutation ΔF1502 in HM, the relevance of these discrete alterations in Ca_V_2.1 channel activity in the context of congenital ataxia remained unclear [44].

Here, we report a second case of congenital ataxia, in a 7 year-old boy, linked to the ΔF1502 α_1A_ mutation, detected by whole-exome sequencing of a parent-child trio. Contrary to the previous reported case (a 13-year-old girl), our patient did not show HM symptoms, although this might be due to its lower age and, therefore, we cannot rule out the possibility that symptoms of HM appear in the future. Besides, our functional study using the physiological permeant ion Ca^2+^ provides robust evidences to consider the ΔF1502 α_1A_ mutation *per se* responsible of such a severe cerebellar phenotype. Thus, ΔF1502 affects several biophysical properties of Ca^2+^-conducting Ca_V_2.1 channels heterologously expressed in mammalian tsA-201 HEK cells: 1) strongly shifts the current activation curve towards hyperpolarized potentials (by ~ 21 mV), allowing significantly higher Ca^2+^ current densities in a range of depolarized voltages with physiological relevance in neurons, even though the mutation reduces maximal Ca^2+^ current density by 50–60%; 2) accelerates activation kinetics and slows deactivation kinetics of Ca_V_2.1 within a wide range of voltage depolarization; 3) slows inactivation kinetic; and 4) shifts voltage-dependent steady-state inactivation to lower voltages (by ~ 28 mV). Despite a favored inactivation by voltage found in the mutant channel, ΔF1502 final effect on Ca_V_2.1 results in a huge Ca^2+^ influx increase in response to stimuli of physiological relevance in neurons, such as either single or trains of action potential-like waveforms (APWs) of different durations. Studies from the homozygous knock-in (KI) mouse carrying the human FHM Ca_V_2.1 R192Q mutation (which decreases voltage threshold of channel activation by ~ 10 mV) reveal that such gain-of-function mutation results in prolonged action potentials in some types of neurons [65]. Our data clearly show that Ca^2+^ influx (Q_Ca_^2+^), through both WT and ΔF1502 Ca_V_2.1 channels, increases with the duration of the action potential-like waveforms (Figs 6, 7C and 8C; S3B and S3C Fig). Therefore, in those neuronal cells where Ca_V_2.1 channels contribute to the shape of action potentials, the ΔF1502-induced increase in Ca^2+^ entry in response to physiological stimuli would be even greater than the one found in our comparisons, stimulating with single or trains of APWs of similar durations both WT and ΔF1502 Ca_V_2.1 channels. Besides, the enhanced Ca^2+^ influx might even be further underestimated (see S3 and S4 Figs) if the observed reduction on maximal Ca^2+^ current density (by 50–60%) is due to aberrant trafficking of the mutant ΔF1502 Ca_V_2.1 channel to the plasma membrane just occurring after heterologous expression in mammalian cells but not in patients’ neurons. Supporting this idea, the previous ΔF1502 functional study only reported a ~ 30% decrease in Ba^2+^ currents through mutant channels expressed in *Xenopus* oocytes [44], whereas we have observed a higher ~ 50–60% decrease in either Ca^2+^ (Figs 4C, 7A and 8A, S1 Fig) or Ba^2+^ (S5 Fig) currents through Ca_V_2.1 ΔF1502 channels expressed in tsA-201 HEK cells. Indeed, a decreased density of functional Ca_V_2.1 channels in the membrane (and a consequent decreased maximal whole-cell Ca^2+^ current density) depending on the cell expression system has been found for most FHM mutants compared to WT [11], making difficult to assess both the consequences of this effect *in vivo* and its real contribution to the associated clinical phenotypes, if any. Still, we cannot rule out the possibility that altered pore gating and/or reduced channel conductance, rather than trafficking problems, are underneath the lower maximal current density through ΔF1502 Ca_V_2.1 channels.

At the structural level, the affected F1502 residue is highly conserved in evolution at the interspecies level as well as among the S6 segments of domains III in the human Ca_V_2 channel family (Fig 3A), indicating functional and/or structural relevance. Based on the crystal structure of the K^+^ channel KcsA, García-Segarra et al. proposed that F1502 is located in the distal half of S6 α helix that faces the cytosolic side, lining the inner pore vestibule of the channel [44]. We have used a different crystal structural model, the bacterial voltage-gated sodium channel (Na_V_Ab), which shares 30% higher homology with human Ca_V_2.1 (T-Coffee ~ 70% score) than the KcsA structural model (T-Coffee ~ 40% score), and found a similar predicted position in the inner pore vestibule for the F1502 residue. Accordingly, Ca_V_2.1 F1502 is located eight residues upstream of a highly conserved isoleucine contributing to the ring of hydrophobic residues which has been suggested to form the internal pore gate in the voltage-gated ion channel superfamily [58]. Our results, along with those obtained from the functional characterization of other Ca_V_2.1 mutations linked to FHM (with or without severe cerebellar symptoms) and located at the distal part of α_1A_ S6 α helices (i.e. V714A, D715E and I1881L, see S2 Fig) [60,63], entail a relevant role of the internal pore mouth in the control of Ca_V_2.1 voltage-dependent gating, affecting both activation and inactivation of the channel. Further studies are required to understand the precise molecular mechanisms by which this pore region influences the operation of voltage sensors in the Ca_V_2.1 channel.

Purkinje cells (PCs) are the sole output from the cerebellar cortex that controls coordination of movement, maintenance of balance and motor learning after integration of multiple sensory and cortical synaptic inputs. Accordingly, defects in PCs firing is thought to be underneath cerebellar dysfunction and ataxia [11]. In this sense, the link between enhanced Ca_V_2.1-mediated Ca^2+^ influx (as described here for the ΔF1502 mutant channel) and permanent or congenital ataxia can be inferred from studies of the homozygous KI mouse carrying the human pathogenic FHM CACNA1A S218L mutation (*Cacna1a* ^S218L/S218L^), which exhibits the main features of the severe clinical syndrome linked to this mutation, including mild permanent cerebellar ataxia [66]. Certainly, it has been shown that the gain-of-function S218L mutation decreases the voltage threshold (by ~ 15 mV) for activation of Ca_V_2.1 channels located in the soma and dendrites of mouse PCs, favoring the generation of somatic action potentials and dendritic Ca^2+^ spikes, and leading to irregular activity patterns. Interestingly, the action potential amplitude, half-width, maximum rising slope, maximum repolarizing slope, decay time constant as well as the basic membrane properties in Purkinje neurons from S218L KI mice were similar to WT cells. Altogether, these results suggest that Ca_V_2.1 gain-of-function mutations affect the initiation threshold of action potentials, but they do not substantially modulate the characteristics of action potentials in murine cerebellar Purkinje neurons [6]. Besides, PCs hyperexcitability in S218L KI mice is further favored by increased Ca^2+^ influx through mutant S218L Ca_V_2.1 channels located at the axon terminals and the subsequent enhancement of neurotransmitter release from excitatory afferent neurons including cerebellar granule cells and neurons in the inferior olive [6]. Independent of whether or not gain-of-function mutations alter the duration of action potentials and/or the firing properties of these afferent neurons (which is unknown at present), the enhanced release of neurotransmitters from their presynaptic terminals is consistent with our observation that single or trains of APWs promote Ca^2+^ entry through gain-of-function mutant Ca_V_2.1 channels. Furthermore, both irregular PCs spiking and ataxic motor performance can be counteracted by activators of hyperpolarizing small-conductance Ca^2+^-activated K^+^ channels (SK channels), suggesting that the S218L-induced hyperexcitability of PCs is indeed the cause of the ataxic phenotype in the mouse model [6]. Similar or even larger effects on cerebellar circuitry function may be produced by other Ca_V_2.1 gain-of-function mutations (as ΔF1502) in humans. Functional alteration of cerebellum circuits by ΔF1502 can explain the fact that our patient presented neurological signs soon after birth and well before neuroimaging was able to disclose the slightest degree of cerebellar atrophy, whose subsequent development may be due to excitotoxic Ca^2+^ signaling causing PCs degeneration, as proposed for spinocerebellar ataxias [67].

Disruption of Ca^2+^ homeostasis leading to higher intracellular Ca^2+^ levels in cerebellar PCs and in synaptic terminals of granule cells has been related with functional and/or developmental defects in the cerebellum, leading to congenital ataxia forms in humans and similar ataxic phenotypes in animal models. Thus, two human missense mutations (S100P and G162R) in the *CA8* gene, encoding carbonic anhydrase 8 (CA8), are linked to mild-to-severe forms of congenital ataxia [68,69]. CA8 is predominantly present in cerebellar PCs, where it interacts with the inositol 1,4,5-trisphosphate receptor type 1 (IP_3_R1). This interaction impairs IP_3_ binding [70,71], therefore modulating the ability of IP_3_R1 to rapidly release Ca^2+^ from the endoplasmatic reticulum (ER). Mutation S100P induces proteasome-mediated degradation with a severe reduction of the level of CA8 protein [68], which is expected to promote ER-mediated intracellular Ca^2+^ signals that in turn may alter PCs synaptic inputs [72,73]. This mutation probably represents a null mutation similar to the 19 bp deletion in the *Ca8* gene of the waddles mouse (a spontaneous animal model with ataxia), in which the lack of detectable Ca8 protein in the cerebellum results in abnormalities in cerebellar synaptic morphology and function [74,75]. More recently, a pivotal role for CA8 during embryonic development has been revealed by the knockdown of CA8 in zebrafish larvae, which results in increased neuronal cell death in the cerebellum and defects in motor and coordination functions, mimicking the CA8-associated ataxic phenotype found in humans and mice [76]. Although no studies are available regarding the effect of mutation G162R on either the function or expression of CA8, a deleterious effect supporting its pathogenicity has been suggested by bioinformatics analysis [69]. A missense mutation (G1107D) in the isoform 3 of the calmodulin-activated plasma membrane Ca^2+^ ATPases (PMCAs) has been also associated to congenital cerebellar ataxia. Functional studies in a heterologous expression system show that mutated pump has reduced ability to extrude Ca^2+^ from the cell. As PMCA3 is highly expressed in the cerebellum, particularly in the excitatory presynaptic terminals of granule cells that make synapsis on PCs, the mutation might lead to higher basal levels of Ca^2+^ and/or altered local Ca^2+^ signaling that affect synaptic efficiency and promote PCs hyperexcitability [77].

In summary, our results highlight the key role of Ca_V_2.1 channel gain-of-function not only in FHM but also in congenital ataxia, making this channel a strong potential drug target for direct, efficient and tailored disease treatment. Indeed, there are pharmacological evidences suggesting that inhibition of channel activity can provide a new therapeutic approach for the treatment of these neurological disorders. Thus, 2,50-di(tertbutyl)-1,4,-benzohydroquinone (BHQ), a synthetic phenolic compound that inhibits sarcoendoplasmic Ca^2+^ ATPases (SERCAs) and with pro-oxidant properties, can also inhibit Ca_V_2.1 voltage-dependent activation to ameliorate gating defects in the channel and subsequently prevent synaptic transmission problems produced by the S218L CACNA1A mutation causing a severe form of FHM with slowly progressive cerebellar ataxia and atrophy in humans [78]. Nevertheless, due to the lack of selectivity on Ca_V_2.1 channels, BHQ itself will not be therapeutically suitable, although modification of its chemical structure may yield small-molecule state-dependent selective inhibitors that could correct the mutation-induced gain-of-function in Ca_V_2.1 activation. Such compounds would greatly improve the chances of developing a personalized medicine approach for *CACNA1A*-linked diseased states of the nervous system. So far, true Ca_V_2.1 selective inhibitors are derived agatoxin family peptides. These peptides are not suitable therapeutic tools: their mode of inhibition can give rise to undesirable side effects and have limited utility for *in vivo* studies.

## Supporting Information

S1 Fig I-V curves corresponding to seals included in the study of ΔF1502 effect on Ca^2+^ influx evoked by single action potential-like waveforms. Average current density-voltage relationships (left) and normalized I-V curves (right) for WT (open circles, n = 14) and ΔF1502 (filled circles, n = 8) Ca_V_2.1 channels expressed in tsA-201 HEK cells. In this series of experiments, maximal Ca^2+^ current density through Ca_V_2.1 channels is still significantly reduced by ΔF1502 (left panel: from -127 ± 23.4 pA/pF (for WT, n = 14) to -63.5 ± 15.3 pA/pF (for ΔF1502, n = 8), P < 0.05, Student’s *t* test) and the significant left-shift induced by ΔF1502 on the Ca_V_2.1 voltage-dependent activation is also observed (right panel: WT V_1/2 act_ = 1.47 ± 0.6 mV (n = 14) *versus* ΔF1502 V_1/2 act_ = -17.2 ± 1.2 mV (n = 8), P < 0.0001, Student’s *t* test).
(TIF)

S2 Fig Ca_V_2.1 missense mutations causing early cerebellar dysfunction compatible with congenital cerebellar ataxia. (A) Location of human missense mutations associated to congenital ataxia and linked (purple circles) or not (purple triangles) to Familial Hemiplegic Migraine (FHM) in the secondary structure of the Ca_V_2.1 α_1A_ channel subunit. For comparison, the location of some pure FHM-linked mutations (red circles) and FHM mutations including progressive ataxia (with cerebellar atrophy in some cases) (cyan circles) are also shown (for details of the corresponding references see S1 Text). The functional consequences of mutations shown in green have been characterized either by heterologous expression of recombinant Ca_V_2.1 channels or by electrophysiological studies of native mutant Ca_V_2.1 channels in neurons from knock-in mice. Note that mutation R1350Q has been also referred as R1349Q and is homologous to the mouse R1252Q allele of the tottering, *Cacna1a* ^*tg*^, mutant series. (B) Sequences of S4 segments at domains I to IV (DI-DIV) of our Ca_V_2.1 α_1A_ channel subunit clone, showing the affected R0 to R5 charged residues (or a neighboring residue) by mutations depicted in (A) (with a similar color pattern to indicate the clinical phenotype linked to the mutation). Positively charged residues in R0 to R5 positions, involved in the movement of the S4 segment in response to voltage, are delineated with a shaded background (gray). Note that R1349Q has been also referred as R1350Q, and R1664Q is equivalent to R1663Q in our α_1A_ clone. (C) Sequences of S6 segments at DI-DIV of our Ca_V_2.1 α_1A_ channel subunit clone, showing the affected residues by mutations illustrated in (A) (with a similar color pattern to indicate the clinical phenotype associated to the mutation). Note that S1799L and I1811L correspond to S1801L and I1812L in our α_1A_ clone, respectively.
(TIF)

S3 Fig Example of expected ΔF1502 effects on Ca^2+^ influx evoked by single action potential-like waveforms (APWs) in the case of unaffected current density. (A) Current density-voltage relationships (left) and the corresponding normalized I-V curves (right) for two particular tsA-201 HEK cells expressing either WT (open circles) or ΔF1502 (filled circles) Ca_V_2.1 channels (maximal Ca^2+^ current density through WT and ΔF1502 Ca_V_2.1 channels are -87.2 pA/pF and -80.8 pA/pF, respectively; V_1/2 act_ values for WT and ΔF1502 Ca_V_2.1 channels are 2.95 mV and -15.17 mV, respectively). (B) Ca^2+^ current traces evoked by APWs of different durations (fast (left panels), medium (central panels) and slow (right panels) (see Materials and Methods for details) obtained from the two tsA-201 HEK cells indicated in panel A (showing similar maximal current densities) expressing either WT (blue traces) or ΔF1502 (red traces) Ca_V_2.1 channels. Dotted lines indicate the zero current level. (C) Values for normalized Ca^2+^ influx (Q_Ca_^2+^) (top panel), time to peak (intermediate panel), and time for Ca^2+^ entry (bottom panel) in response to the above-mentioned APWs obtained from these two cells expressing either WT (blue bars) or ΔF1502 (red bars) Ca_V_2.1 channels.
(TIF)

S4 Fig Example of expected ΔF1502 effects on Ca^2+^ influx evoked by a 42 Hz train of 2 ms action potential-like waveforms (APWs) in the case of unaffected current density. (A) Current density-voltage relationships (left) and the corresponding normalized I-V curves (right) for two particular tsA-201 HEK cells expressing either WT (open circles) or ΔF1502 (filled circles) Ca_V_2.1 channels (maximal Ca^2+^ current density through WT and ΔF1502 Ca_V_2.1 channels are -87.8 pA/pF and -79.51 pA/pF, respectively; V_1/2 act_ values for WT and ΔF1502 Ca_V_2.1 channels are 1.56 mV and -15.87 mV, respectively). (B) Ca^2+^ current traces evoked by every 200^th^ pulse of a 42 Hz train of medium (2 ms) APWs (see Materials and Methods for details) obtained from the two tsA-201 HEK cells indicated in panel A (showing similar maximal current densities) expressing either WT (left) or ΔF1502 (right) Ca_V_2.1 channels. Dotted lines indicate the zero current level. (C) Values for Ca^2+^ influx normalized by cell size (Q_Ca_^2+^) in response to every 5^th^ pulse of a 42 Hz train of medium (2 ms) APWs, obtained from these two cells expressing either WT (blue symbols) or ΔF1502 (red symbols) Ca_V_2.1 channels.
(TIF)

S5 Fig Effect of ΔF1502 on Ca_V_2.1-mediated Ba^2+^ currents: reduction in IBa2+ density and ∼11 mV left-shift in the voltage dependence of activation. Average current density-voltage relationships normalized by the absolute maximal value (left) and I-V curves normalized by the peak current obtained in each recording (right), for WT (open circles, n = 14) and ΔF1502 (filled circles, n = 9) Ca_V_2.1 channels expressed in tsA-201 HEK cells and using 2.5 mM BaCl_2_ instead of CaCl_2_ in the extracellular recording solution. ΔF1502 induces a ~ 61% significant reduction of maximal Ba^2+^ currents through Ca_V_2.1 channels (P < 0.01, Mann-Whitney U-test), as found for maximal Ca^2+^ currents (see Figs 4C, 7A and 8A, S1 Fig). As previously reported [44], when using Ba^2+^ as the charge carrier, ΔF1502 only induces a significant ~ 11 mV left-shift on the Ca_V_2.1 voltage-dependent activation (right panel: WT V_1/2 act_ = -8.23 ± 1.02 mV (n = 14) *versus* ΔF1502 V_1/2 act_ = -18.95 ± 1.8 mV (n = 9), P < 0.001, Mann-Whitney U-test).
(TIF)

S1 Table Average peak inward Ca^2+^ current densities (pA/pF) through either WT or ΔF1502 Ca_V_2.1 channels, elicited by 20 ms depolarizing pulses from a holding of -80 mV to the indicated voltages. Data are presented as the means ± S.E.M. For statistical comparison we used the Mann-Whitney U-test.
(DOCX)

S2 Table Average activation kinetics of WT and ΔF1502 Ca_V_2.1 channels at the indicated depolarizing voltages. Data are presented as the means ± S.E.M. The Mann-Whitney U-test was employed for statistical comparison.
(DOCX)

S3 Table Average deactivation kinetics of WT and ΔF1502 Ca_V_2.1 channels at the indicated voltages. Data are presented as the means ± S.E.M. For statistical comparison we used the Mann-Whitney U-test.
(DOCX)

S1 Text References for S2 Fig. (DOCX)
