# Supplementary figures and images for: A Single Amino Acid Deletion (ΔF1502) in the S6 Segment of CaV2.1 Domain III Associated with Congenital Ataxia Increases Channel Activity and Promotes Ca2+ Influx
Source: PLoS One. 2015 Dec 30;10(12):e0146035. doi: 10.1371/journal.pone.0146035 (PMC4696675; doi:10.1371/journal.pone.0146035)

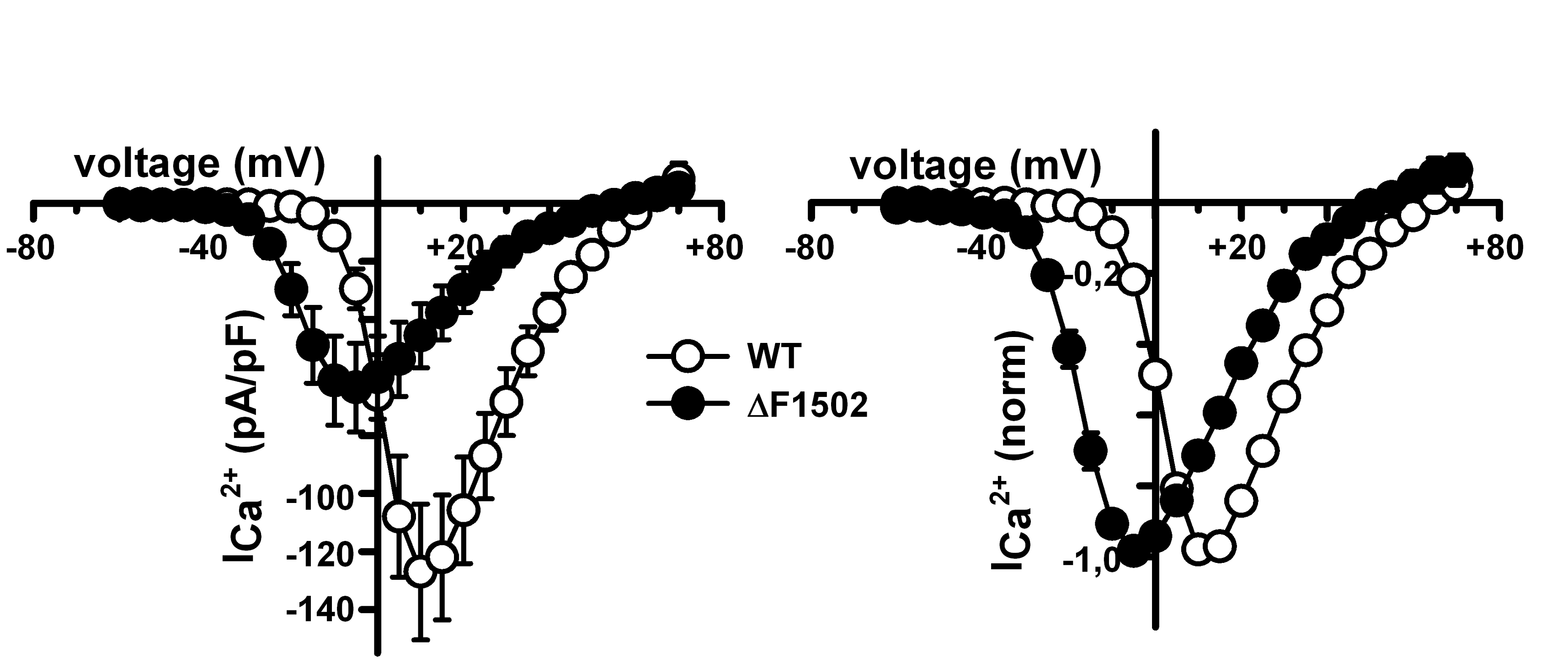

Supplement: S1 Fig — Average current density-voltage relationships (left) and normalized I-V curves (right) for WT (open circles, n = 14) and ΔF1502 (filled circles, n = 8) CaV2.1 channels expressed in tsA-201 HEK cells. In this series of experiments, maximal Ca2+ current density through CaV2.1 channels is still significantly reduced by ΔF1502 (left panel: from -127 ± 23.4 pA/pF (for WT, n = 14) to -63.5 ± 15.3 pA/pF (for ΔF1502, n = 8), P < 0.05, Student’s t test) and the significant left-shift induced by ΔF1502 on the CaV2.1 voltage-dependent activation is also observed (right panel: WT V1/2 act = 1.47 ± 0.6 mV (n = 14) versus ΔF1502 V1/2 act = -17.2 ± 1.2 mV (n = 8), P < 0.0001, Student’s t test). (TIF) [file pone.0146035.s001.tif]

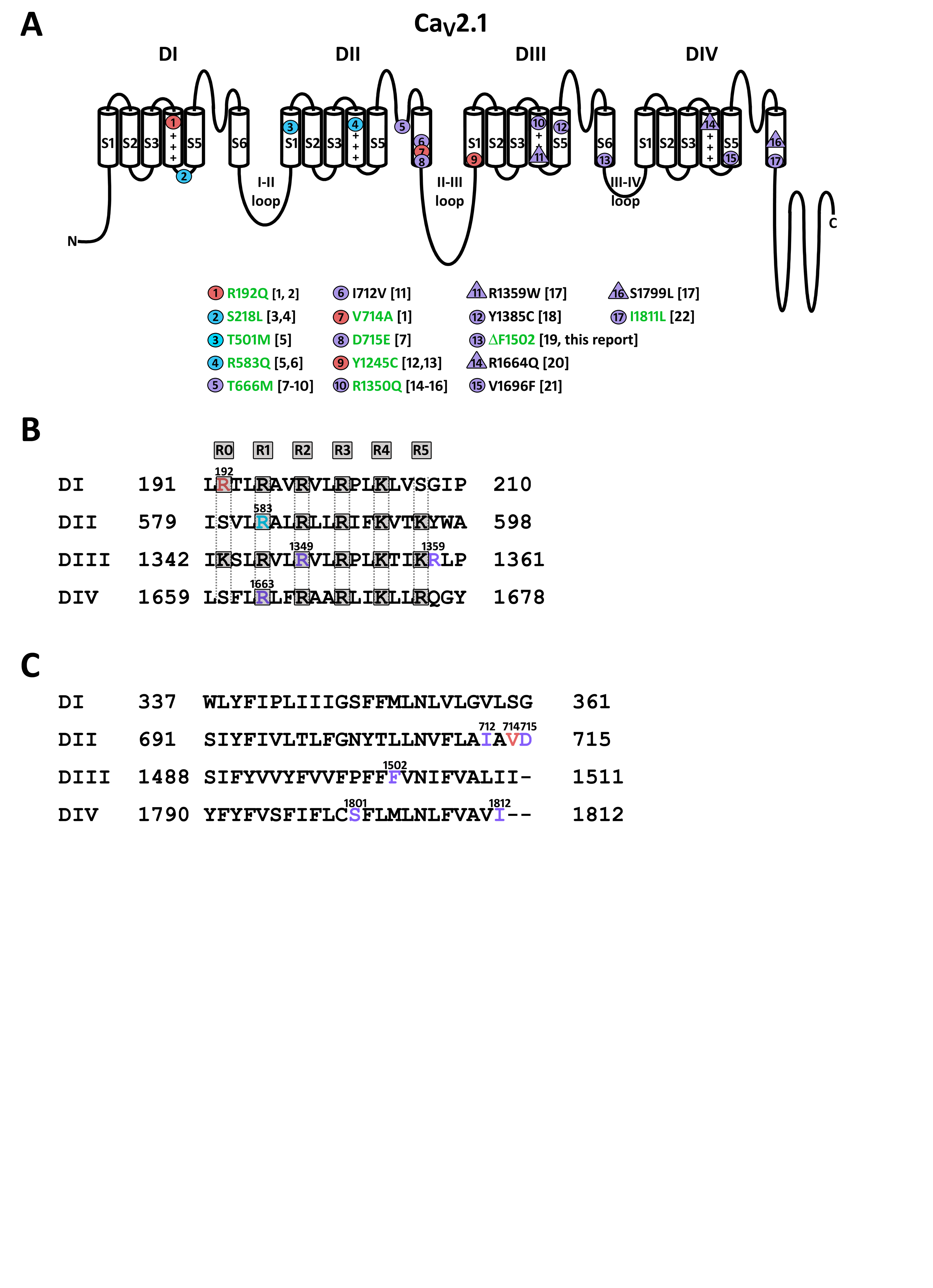

Supplement: S2 Fig — (A) Location of human missense mutations associated to congenital ataxia and linked (purple circles) or not (purple triangles) to Familial Hemiplegic Migraine (FHM) in the secondary structure of the CaV2.1 α1A channel subunit. For comparison, the location of some pure FHM-linked mutations (red circles) and FHM mutations including progressive ataxia (with cerebellar atrophy in some cases) (cyan circles) are also shown (for details of the corresponding references see S1 Text). The functional consequences of mutations shown in green have been characterized either by heterologous expression of recombinant CaV2.1 channels or by electrophysiological studies of native mutant CaV2.1 channels in neurons from knock-in mice. Note that mutation R1350Q has been also referred as R1349Q and is homologous to the mouse R1252Q allele of the tottering, Cacna1a tg, mutant series. (B) Sequences of S4 segments at domains I to IV (DI-DIV) of our CaV2.1 α1A channel subunit clone, showing the affected R0 to R5 charged residues (or a neighboring residue) by mutations depicted in (A) (with a similar color pattern to indicate the clinical phenotype linked to the mutation). Positively charged residues in R0 to R5 positions, involved in the movement of the S4 segment in response to voltage, are delineated with a shaded background (gray). Note that R1349Q has been also referred as R1350Q, and R1664Q is equivalent to R1663Q in our α1A clone. (C) Sequences of S6 segments at DI-DIV of our CaV2.1 α1A channel subunit clone, showing the affected residues by mutations illustrated in (A) (with a similar color pattern to indicate the clinical phenotype associated to the mutation). Note that S1799L and I1811L correspond to S1801L and I1812L in our α1A clone, respectively. (TIF) [file pone.0146035.s002.tif]

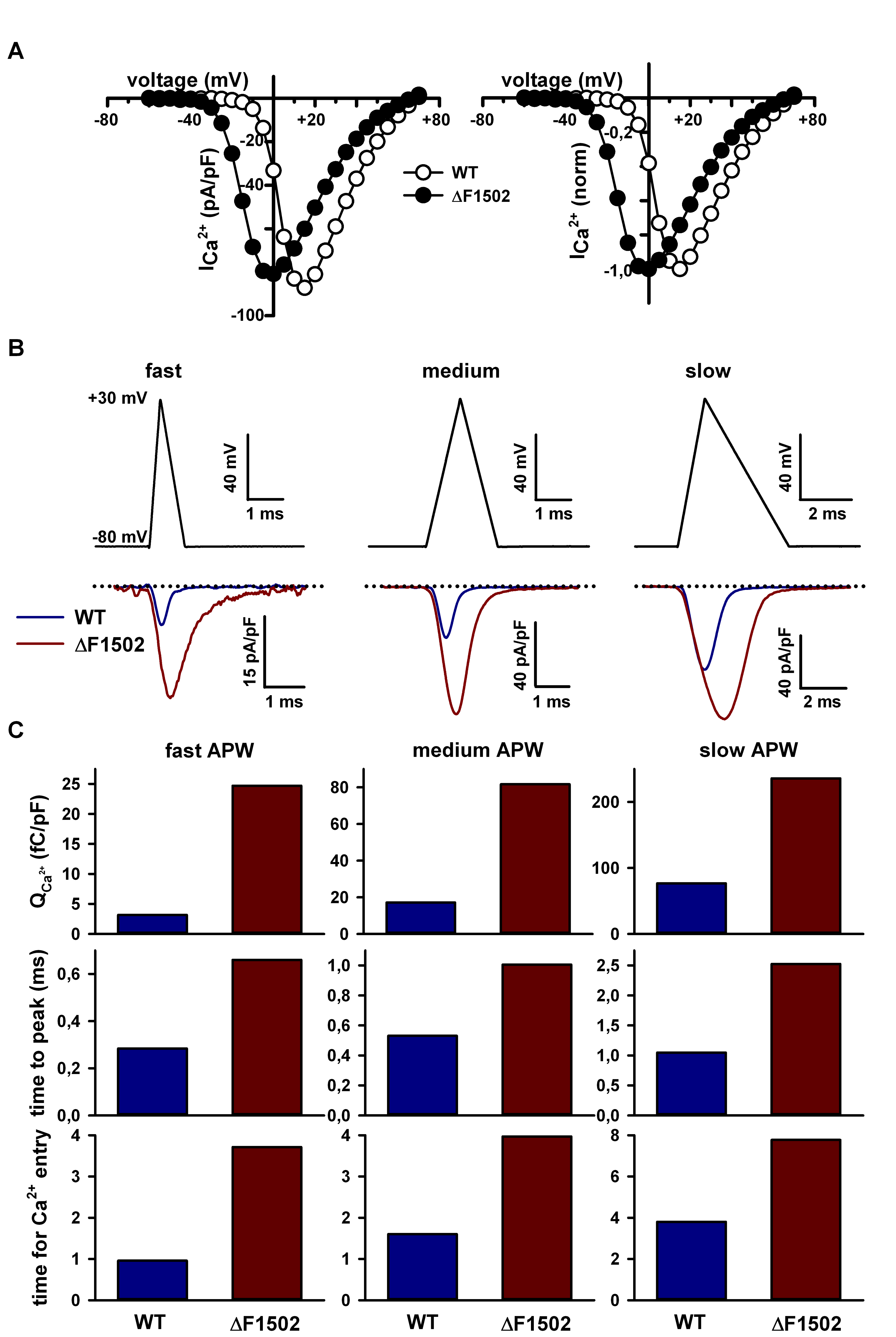

Supplement: S3 Fig — (A) Current density-voltage relationships (left) and the corresponding normalized I-V curves (right) for two particular tsA-201 HEK cells expressing either WT (open circles) or ΔF1502 (filled circles) CaV2.1 channels (maximal Ca2+ current density through WT and ΔF1502 CaV2.1 channels are -87.2 pA/pF and -80.8 pA/pF, respectively; V1/2 act values for WT and ΔF1502 CaV2.1 channels are 2.95 mV and -15.17 mV, respectively). (B) Ca2+ current traces evoked by APWs of different durations (fast (left panels), medium (central panels) and slow (right panels) (see Materials and Methods for details) obtained from the two tsA-201 HEK cells indicated in panel A (showing similar maximal current densities) expressing either WT (blue traces) or ΔF1502 (red traces) CaV2.1 channels. Dotted lines indicate the zero current level. (C) Values for normalized Ca2+ influx (QCa 2+) (top panel), time to peak (intermediate panel), and time for Ca2+ entry (bottom panel) in response to the above-mentioned APWs obtained from these two cells expressing either WT (blue bars) or ΔF1502 (red bars) CaV2.1 channels. (TIF) [file pone.0146035.s003.tif]

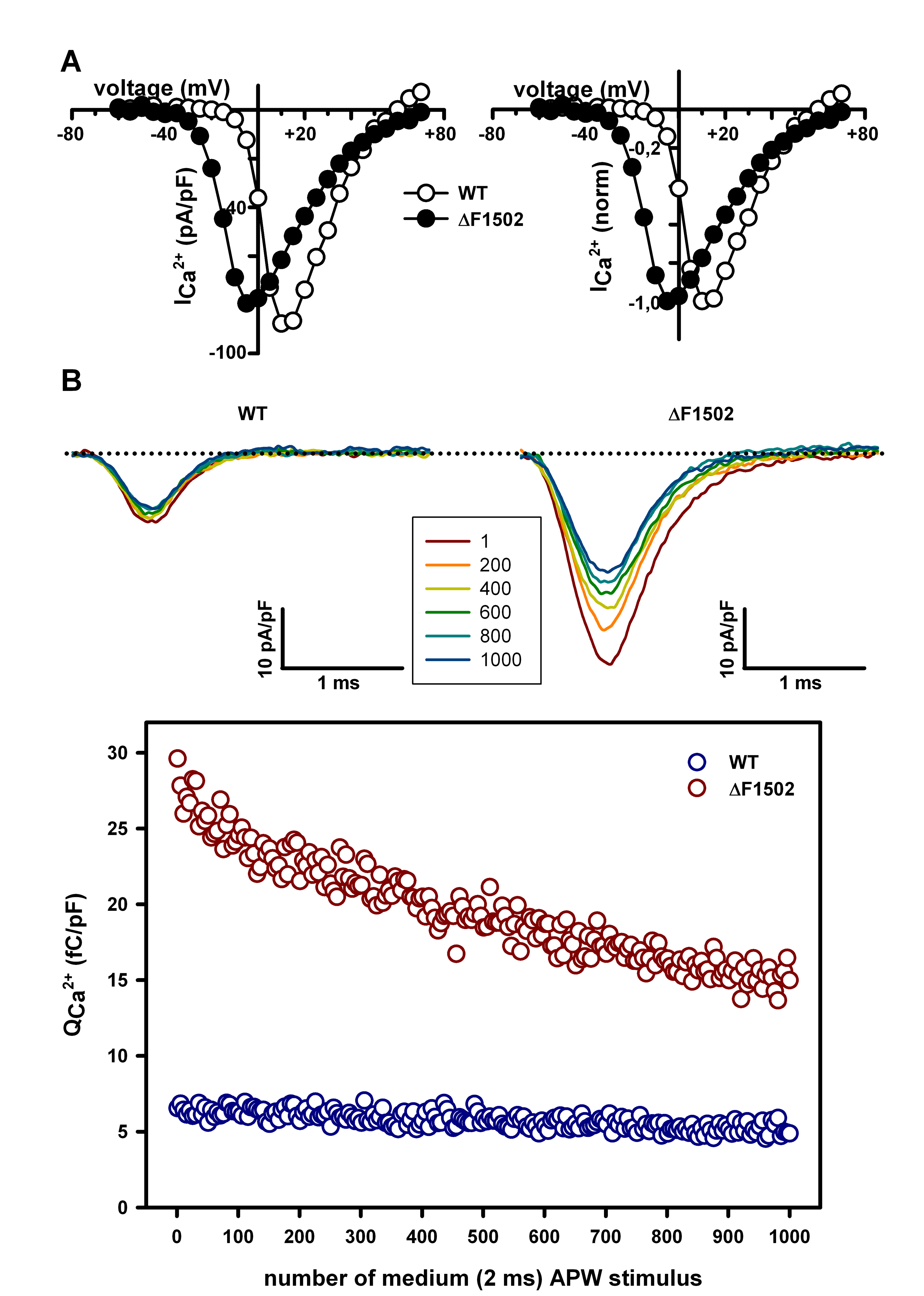

Supplement: S4 Fig — (A) Current density-voltage relationships (left) and the corresponding normalized I-V curves (right) for two particular tsA-201 HEK cells expressing either WT (open circles) or ΔF1502 (filled circles) CaV2.1 channels (maximal Ca2+ current density through WT and ΔF1502 CaV2.1 channels are -87.8 pA/pF and -79.51 pA/pF, respectively; V1/2 act values for WT and ΔF1502 CaV2.1 channels are 1.56 mV and -15.87 mV, respectively). (B) Ca2+ current traces evoked by every 200th pulse of a 42 Hz train of medium (2 ms) APWs (see Materials and Methods for details) obtained from the two tsA-201 HEK cells indicated in panel A (showing similar maximal current densities) expressing either WT (left) or ΔF1502 (right) CaV2.1 channels. Dotted lines indicate the zero current level. (C) Values for Ca2+ influx normalized by cell size (QCa 2+) in response to every 5th pulse of a 42 Hz train of medium (2 ms) APWs, obtained from these two cells expressing either WT (blue symbols) or ΔF1502 (red symbols) CaV2.1 channels. (TIF) [file pone.0146035.s004.tif]

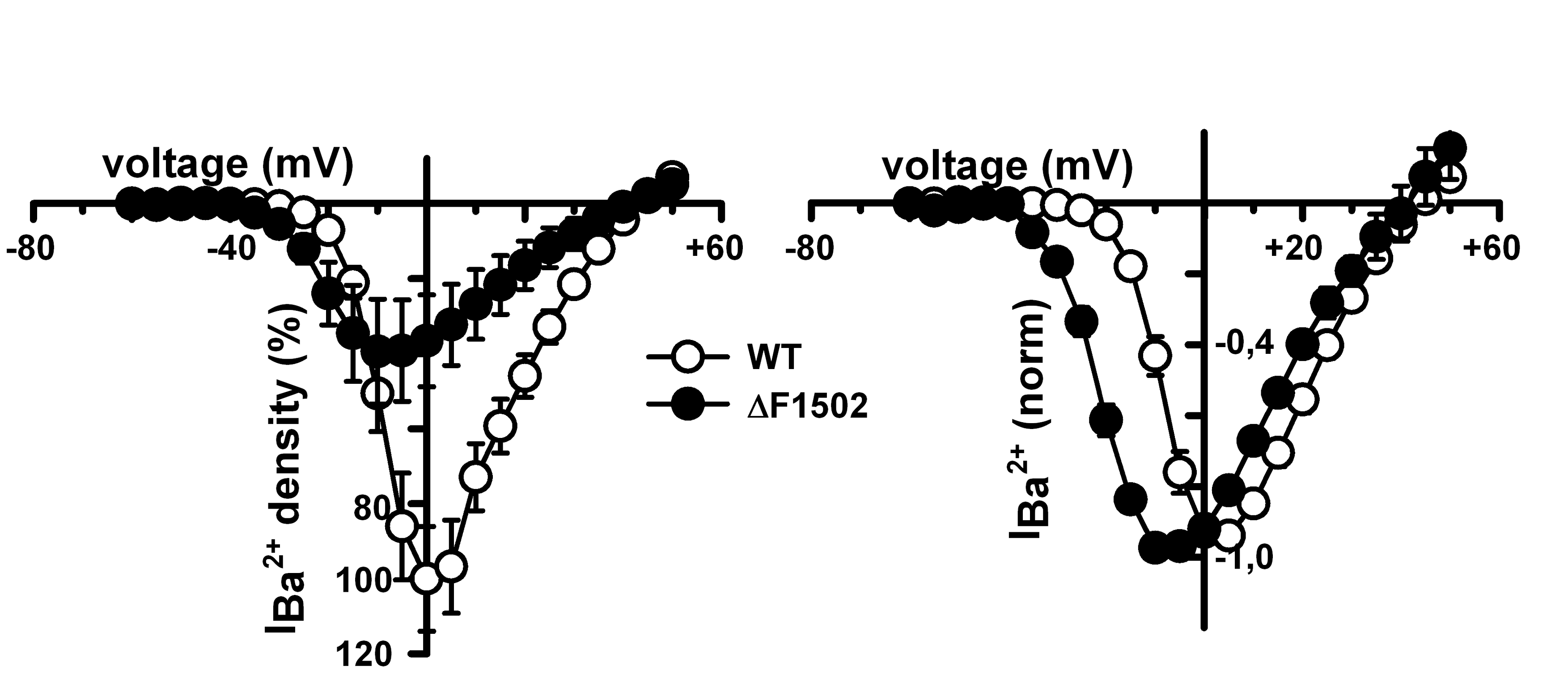

Supplement: S5 Fig — Average current density-voltage relationships normalized by the absolute maximal value (left) and I-V curves normalized by the peak current obtained in each recording (right), for WT (open circles, n = 14) and ΔF1502 (filled circles, n = 9) CaV2.1 channels expressed in tsA-201 HEK cells and using 2.5 mM BaCl2 instead of CaCl2 in the extracellular recording solution. ΔF1502 induces a ~ 61% significant reduction of maximal Ba2+ currents through CaV2.1 channels (P < 0.01, Mann-Whitney U-test), as found for maximal Ca2+ currents (see Figs 4C, 7A and 8A, S1 Fig). As previously reported [44], when using Ba2+ as the charge carrier, ΔF1502 only induces a significant ~ 11 mV left-shift on the CaV2.1 voltage-dependent activation (right panel: WT V1/2 act = -8.23 ± 1.02 mV (n = 14) versus ΔF1502 V1/2 act = -18.95 ± 1.8 mV (n = 9), P < 0.001, Mann-Whitney U-test). (TIF) [file pone.0146035.s005.tif]
