# Supplementary material for: A Single Amino Acid Deletion (ΔF1502) in the S6 Segment of CaV2.1 Domain III Associated with Congenital Ataxia Increases Channel Activity and Promotes Ca2+ Influx
Source: PLoS One. 2015 Dec 30;10(12):e0146035. doi: 10.1371/journal.pone.0146035 (PMC4696675; doi:10.1371/journal.pone.0146035)
Supplement: S1 Table — Data are presented as the means ± S.E.M. For statistical comparison we used the Mann-Whitney U-test. (DOCX) [file pone.0146035.s006.docx]

**S1 Table.**

| **depolarizing voltage (mV)** | **WT peak I_Ca_^2+^ (pA/pF)** | **ΔF1502 peak I_Ca_^2+^ (pA/pF)** | **P value** |
| --- | --- | --- | --- |
| -40 | 0.39 ± 0.1 (n = 27) | 0.92 ± 0.2 (n = 19) | P < 0.05 |
| -35 | 0.36 ± 0.08 (n = 27) | 1.34 ± 0.39 (n = 19) | P < 0.05 |
| -30 | 0.44 ± 0.13 (n = 27) | 3.39 ± 0.9 (n = 19) | P < 0.0001 |
| -25 | 0.7 ± 0.15 (n = 27) | 7.92 ± 2.26 (n = 19) | P < 0.0001 |
| -20 | 0.79 ± 0.26 (n = 27) | 16.46 ± 4.55 (n = 19) | P < 0.0001 |
| -15 | 2.03 ± 0.43 (n = 27) | 26.63 ± 6.98 (n = 19) | P < 0.0001 |
| -10 | 5.55 ± 1.2 (n = 27) | 33.3 ± 8.42 (n = 19) | P < 0.0001 |
| -5 | 14.47 ± 3.19 (n = 27) | 35.16 ± 8.47 (n = 19) | P < 0.01 |
