# Supplementary material for: A Single Amino Acid Deletion (ΔF1502) in the S6 Segment of CaV2.1 Domain III Associated with Congenital Ataxia Increases Channel Activity and Promotes Ca2+ Influx
Source: PLoS One. 2015 Dec 30;10(12):e0146035. doi: 10.1371/journal.pone.0146035 (PMC4696675; doi:10.1371/journal.pone.0146035)
Supplement: S2 Table — Data are presented as the means ± S.E.M. The Mann-Whitney U-test was employed for statistical comparison. (DOCX) [file pone.0146035.s007.docx]

**S2 Table.**

| **depolarizing voltage (mV)** | **WT *τ*_activation_ (ms)** | **ΔF1502 *τ*_activation_ (ms)** | **P value** |
| --- | --- | --- | --- |
| 0 | 2.15 ± 0.13 (n = 27) | 1.52 ± 0.08 (n = 19) | P < 0.001 |
| +5 | 2.48 ± 0.2 (n = 27) | 1.22 ± 0.08 (n = 19) | P < 0.0001 |
| +10 | 2.3 ± 0.24 (n = 27) | 1.04 ± 0.06 (n = 19) | P < 0.0001 |
| +15 | 1.75 ± 0.16 (n = 27) | 0.78 ± 0.05 (n = 19) | P < 0.0001 |
| +20 | 1.37 ± 0.11 (n = 27) | 0.7 ± 0.04 (n = 19) | P < 0.0001 |
| +25 | 1.12 ± 0.08 (n = 27) | 0.61 ± 0.05 (n = 19) | P < 0.0001 |
| +30 | 0.92 ± 0.06 (n = 27) | 0.54 ± 0.05 (n = 11) | P < 0.001 |
| +35 | 0.82 ± 0.05 (n = 27) | 0.51 ± 0.04 (n = 10) | P < 0.001 |
| +40 | 0.71 ± 0.05 (n = 24) | 0.41 ± 0.05 (n = 9) | P < 0.05 |
| +45 | 0.62 ± 0.05 (n = 23) | 0.37 ± 0.03 (n = 8) | P < 0.001 |
| +50 | 0.55 ± 0.04 (n = 23) | 0.28 ± 0.03 (n = 5) | P < 0.001 |
| +55 | 0.52 ± 0.05 (n = 21) | 0.29 ± 0.07 (n = 5) | P < 0.05 |
