# Supplementary material for: A Single Amino Acid Deletion (ΔF1502) in the S6 Segment of CaV2.1 Domain III Associated with Congenital Ataxia Increases Channel Activity and Promotes Ca2+ Influx
Source: PLoS One. 2015 Dec 30;10(12):e0146035. doi: 10.1371/journal.pone.0146035 (PMC4696675; doi:10.1371/journal.pone.0146035)
Supplement: S3 Table — Data are presented as the means ± S.E.M. For statistical comparison we used the Mann-Whitney U-test. (DOCX) [file pone.0146035.s008.docx]

**S3 Table.**

| **depolarizing voltage (mV)** | **WT *τ*_deactivation_ (ms)** | **ΔF1502 *τ*_deactivation_ (ms)** | **P value** |
| --- | --- | --- | --- |
| -80 | 0.27 ± 0.03 (n = 9) | 0.54 ± 0.04 (n = 14) | P < 0.0001 |
| -75 | 0.27 ± 0.03 (n = 9) | 0.57 ± 0.04 (n = 14) | P < 0.0001 |
| -70 | 0.28 ± 0.03 (n = 9) | 0.67 ± 0.07 (n = 14) | P < 0.0001 |
| -65 | 0.29 ± 0.03 (n = 9) | 0.71 ± 0.05 (n = 14) | P < 0.0001 |
| -60 | 0.31 ± 0.05 (n = 9) | 0.84 ± 0.04 (n = 14) | P < 0.0001 |
| -55 | 0.32 ± 0.05 (n = 9) | 1.03 ± 0.08 (n = 14) | P < 0.0001 |
| -50 | 0.34 ± 0.05 (n = 9) | 1.26 ± 0.07 (n = 14) | P < 0.0001 |
| -45 | 0.37 ± 0.06 (n = 9) | 1.52 ± 0.09 (n = 14) | P < 0.0001 |
| -40 | 0.42 ± 0.08 (n = 9) | 2.07 ± 0.14 (n = 14) | P < 0.0001 |
| -35 | 0.46 ± 0.09 (n = 9) | 2.63 ± 0.18 (n = 14) | P < 0.0001 |
| -30 | 0.5 ± 0.08 (n = 9) | 3.29 ± 0.21 (n = 14) | P < 0.0001 |
| -25 | 0.61 ± 0.11 (n = 9) | 4.63 ± 0.37 (n = 14) | P < 0.0001 |
| -20 | 0.75 ± 0.12 (n = 9) | 5.55 ± 0.44 (n = 12) | P < 0.0001 |
