# Supplementary material for: A Single Amino Acid Deletion (ΔF1502) in the S6 Segment of CaV2.1 Domain III Associated with Congenital Ataxia Increases Channel Activity and Promotes Ca2+ Influx
Source: PLoS One. 2015 Dec 30;10(12):e0146035. doi: 10.1371/journal.pone.0146035 (PMC4696675; doi:10.1371/journal.pone.0146035)
Supplement: S1 Text — (DOCX) [file pone.0146035.s009.docx]

**S1 Text. References for S2 Fig.**

1. [Ophoff RA](http://www.ncbi.nlm.nih.gov/pubmed/?term=Ophoff%20RA%5BAuthor%5D&cauthor=true&cauthor_uid=8898206), [Terwindt GM](http://www.ncbi.nlm.nih.gov/pubmed/?term=Terwindt%20GM%5BAuthor%5D&cauthor=true&cauthor_uid=8898206), [Vergouwe MN](http://www.ncbi.nlm.nih.gov/pubmed/?term=Vergouwe%20MN%5BAuthor%5D&cauthor=true&cauthor_uid=8898206), [van Eijk R](http://www.ncbi.nlm.nih.gov/pubmed/?term=van%20Eijk%20R%5BAuthor%5D&cauthor=true&cauthor_uid=8898206), [Oefner PJ](http://www.ncbi.nlm.nih.gov/pubmed/?term=Oefner%20PJ%5BAuthor%5D&cauthor=true&cauthor_uid=8898206), [Hoffman SM](http://www.ncbi.nlm.nih.gov/pubmed/?term=Hoffman%20SM%5BAuthor%5D&cauthor=true&cauthor_uid=8898206) et al. Familial hemiplegic migraine and episodic ataxia type-2 are caused by mutations in the Ca^2+^ channel gene *CACNL1A4*. [Cell.](http://www.ncbi.nlm.nih.gov/pubmed/?term=Cell+87%2C+543%E2%80%93552) 1996;87: 543-52.
2. Pietrobon D. Familial hemiplegic migraine. Neurotherapeutics. 2007;4: 274-284
3. Fitzsimons RB, Wolfenden WH. Migraine coma: meningitic migraine with cerebral oedema associated with a new form of autosomal dominant cerebellar ataxia. Brain. 1985;108: 555-577.
4. Kors EE, Terwindt GM, Vermeulen FL, Fitzsimons RB, Jardine PE, Heywood P, et al. Delayed cerebral edema and fatal coma after minor head trauma: role of the *CACNA1A* calcium channel subunit gene and relationship with familial hemiplegic migraine. Ann Neurol. 2001;49: 753-760.
5. Carreño O, Corominas R, Serra SA, Sintas C, Fernández-Castillo N, Vila-Pueyo M, et al. Screening of *CACNA1A* and *ATP1A2* genes in hemiplegic migraine: clinical, genetic, and functional studies. Mol Genet & Genomic Med. 2013;1: 206-222.
6. [Battistini S](http://www.ncbi.nlm.nih.gov/pubmed/?term=Battistini%20S%5BAuthor%5D&cauthor=true&cauthor_uid=10408534), [Stenirri S](http://www.ncbi.nlm.nih.gov/pubmed/?term=Stenirri%20S%5BAuthor%5D&cauthor=true&cauthor_uid=10408534), [Piatti M](http://www.ncbi.nlm.nih.gov/pubmed/?term=Piatti%20M%5BAuthor%5D&cauthor=true&cauthor_uid=10408534), [Gelfi C](http://www.ncbi.nlm.nih.gov/pubmed/?term=Gelfi%20C%5BAuthor%5D&cauthor=true&cauthor_uid=10408534), [Righetti PG](http://www.ncbi.nlm.nih.gov/pubmed/?term=Righetti%20PG%5BAuthor%5D&cauthor=true&cauthor_uid=10408534), [Rocchi R](http://www.ncbi.nlm.nih.gov/pubmed/?term=Rocchi%20R%5BAuthor%5D&cauthor=true&cauthor_uid=10408534), et al. A new CACNA1A gene mutation in acetazolamide-responsive familial hemiplegic migraine and ataxia. [Neurology.](http://www.ncbi.nlm.nih.gov/pubmed/10408534) 1999;53: 38-43.
7. Ducros A, Denier C, Joutel A, Vahedi K, Michel A, Darcel F, et al. Recurrence of the T666M calcium channel *CACNA1A* gene mutation in familial hemiplegic migraine with progressive cerebellar ataxia. Am J Hum Genet. 1999;64:89-98.
8. Wada T, Kobayashi N, Takahashi Y, Aoki T, Watanabe T, Saitoh S. Wide clinical variability in a family with a CACNA1A T666M mutation. Hemiplegic coma and progressive ataxia. Pediatr Neurol. 2002;26: 47-50.
9. Freilinger T, Bohe M, Wegener B, Müller-Myhsok B, DichgansM, Knoblauch H. Expansion of the phenotypic spectrum of the *CACNA1A* T666M mutation: a family with familial hemiplegic migraine type 1, cerebellar atrophy and mental retardation. Cephalalgia. 2008;28: 403-407.
10. Naik S, Pohl K, Malik M, Siddiqui A, Josifova D. Early-onset cerebellar atrophy associated with mutation in the *CACNA1A* gene. Pediatr Neurol. 2011;45: 328-330.
11. Guerin AA, Feigenbaum A, Donner EJ, Yoon G. Stepwise Developmental regression associated with novel *CACNA1A* mutation. [Pediatr Neurol.](http://www.ncbi.nlm.nih.gov/pubmed/?term=Stepwise+Developmental+Regression+Associated+With+Novel+CACNA1A+Mutation) 2008;39: 363-4.
12. Cuenca-León E, Corominas R, Fernàndez-Castillo N, Volpini V, del Toro M, Roig M, et al. Genetic analysis of 27 Spanish patients with hemiplegic migraine, basilar-type migraine and childhood periodic syndromes. Cephalalgia. 2008;28: 1039-1047.
13. Serra SA, Fernàndez-Castillo N, Macaya A, Cormand B, Valverde MA, Fernández-Fernández JM. The Hemiplegic Migraine associated Y1245C mutation in CACNA1A results in a gain of channel function due to its effect on the voltage sensor and G-protein mediated inhibition. Pflügers Arch - Eur J Physiol. 2009;458: 489-502.
14. [Blumkin L](http://www.ncbi.nlm.nih.gov/pubmed/?term=Blumkin%20L%5BAuthor%5D&cauthor=true&cauthor_uid=20097664), [Michelson M](http://www.ncbi.nlm.nih.gov/pubmed/?term=Michelson%20M%5BAuthor%5D&cauthor=true&cauthor_uid=20097664), [Leshinsky-Silver E](http://www.ncbi.nlm.nih.gov/pubmed/?term=Leshinsky-Silver%20E%5BAuthor%5D&cauthor=true&cauthor_uid=20097664), [Kivity S](http://www.ncbi.nlm.nih.gov/pubmed/?term=Kivity%20S%5BAuthor%5D&cauthor=true&cauthor_uid=20097664), [Lev D](http://www.ncbi.nlm.nih.gov/pubmed/?term=Lev%20D%5BAuthor%5D&cauthor=true&cauthor_uid=20097664), [Lerman-Sagie T](http://www.ncbi.nlm.nih.gov/pubmed/?term=Lerman-Sagie%20T%5BAuthor%5D&cauthor=true&cauthor_uid=20097664). Congenital ataxia, mental retardation, and dyskinesia associated with a novel CACNA1A mutation. [J Child Neurol.](http://www.ncbi.nlm.nih.gov/pubmed/?term=r1350Q+AND+cacna1a) 2010;25:892-7.
15. [Knierim E](http://www.ncbi.nlm.nih.gov/pubmed/?term=Knierim%20E%5BAuthor%5D&cauthor=true&cauthor_uid=21183743), [Leisle L](http://www.ncbi.nlm.nih.gov/pubmed/?term=Leisle%20L%5BAuthor%5D&cauthor=true&cauthor_uid=21183743), [Wagner C](http://www.ncbi.nlm.nih.gov/pubmed/?term=Wagner%20C%5BAuthor%5D&cauthor=true&cauthor_uid=21183743), [Weschke B](http://www.ncbi.nlm.nih.gov/pubmed/?term=Weschke%20B%5BAuthor%5D&cauthor=true&cauthor_uid=21183743), [Lucke B](http://www.ncbi.nlm.nih.gov/pubmed/?term=Lucke%20B%5BAuthor%5D&cauthor=true&cauthor_uid=21183743), [Bohner G](http://www.ncbi.nlm.nih.gov/pubmed/?term=Bohner%20G%5BAuthor%5D&cauthor=true&cauthor_uid=21183743), et al. Recurrent stroke due to a novel voltage sensor mutation in Cav2.1 responds to verapamil. [Stroke.](http://www.ncbi.nlm.nih.gov/pubmed/?term=Recurrent+Stroke+Due+to+a+Novel+Voltage+Sensor+Mutation) 2011;42: e14-7.
16. Miki T, Zwingman TA, Wakamori M, Lutz CM, Cook SA, Hosford DA, et al. Two novel alleles of tottering with distinct Ca_V_2.1 calcium channel neuropathologies. Neuroscience. 2008;155:31-44.
17. Ohba C, Osaka H, Iai M, Yamashita S, Suzuki Y, Aida N, et al. Diagnostic utility of whole exome sequencing in patients showing cerebellar and/or vermis atrophy in childhood. Neurogenetics. 2013;14: 225-232.
18. Vahedi K, Denier C, Ducros A, Bousson V, Levy C, Chabriat H, et al. *CACNA1A* gene de novo mutation causing hemiplegic migraine, coma, and cerebellar atrophy. Neurology. 2000;55: 1040-1042.
19. García-Segarra N, Gautschi I, Mittaz-Crettol L, Kallay Zetchi C, Al-Qusairi L, Van Bemmelen MX, et al. Congenital ataxia and hemiplegic migraine with cerebral edema associated with a novel gain of function mutation in the calcium channel CACNA1A. J Neurol Sci. 2014;342: 69-78.
20. Tonelli A, D’Angelo MG, Salati R, Villa L, Germinasi C, Frattini T, et al. Early onset, non fluctuating spinocerebellar ataxia and a novel missense mutation in *CACNA1A* gene. Journal of the Neurological Sciences. 2006;241: 13-17.
21. de Vries B, Stam AH, Beker F, van den Maagdenberg AMJM, [Vanmolkot KR](http://www.ncbi.nlm.nih.gov/pubmed/?term=Vanmolkot%20KR%5BAuthor%5D&cauthor=true&cauthor_uid=18498393), [Laan L](http://www.ncbi.nlm.nih.gov/pubmed/?term=Laan%20L%5BAuthor%5D&cauthor=true&cauthor_uid=18498393), [Ginjaar IB](http://www.ncbi.nlm.nih.gov/pubmed/?term=Ginjaar%20IB%5BAuthor%5D&cauthor=true&cauthor_uid=18498393), et al. *CACNA1A* mutation linking hemiplegic migraine and alternating hemiplegia of childhood. Cephalalgia. 2008;28: 887-891.
22. [Terwindt GM](http://www.ncbi.nlm.nih.gov/pubmed/?term=Terwindt%20GM%5BAuthor%5D&cauthor=true&cauthor_uid=9566402), [Ophoff RA](http://www.ncbi.nlm.nih.gov/pubmed/?term=Ophoff%20RA%5BAuthor%5D&cauthor=true&cauthor_uid=9566402), [Haan J](http://www.ncbi.nlm.nih.gov/pubmed/?term=Haan%20J%5BAuthor%5D&cauthor=true&cauthor_uid=9566402), [Vergouwe MN](http://www.ncbi.nlm.nih.gov/pubmed/?term=Vergouwe%20MN%5BAuthor%5D&cauthor=true&cauthor_uid=9566402), [van Eijk R](http://www.ncbi.nlm.nih.gov/pubmed/?term=van%20Eijk%20R%5BAuthor%5D&cauthor=true&cauthor_uid=9566402), [Frants RR](http://www.ncbi.nlm.nih.gov/pubmed/?term=Frants%20RR%5BAuthor%5D&cauthor=true&cauthor_uid=9566402), [et](http://www.ncbi.nlm.nih.gov/pubmed/?term=Ferrari%20MD%5BAuthor%5D&cauthor=true&cauthor_uid=9566402) al. Variable clinical expression of mutations in the P/Q-type calcium channel gene in familial hemiplegic migraine. Dutch Migraine Genetics Research Group. [Neurology.](http://www.ncbi.nlm.nih.gov/pubmed/9566402) 1998;50: 1105-1110.
